# Evolution of the Human Chromosome 13 Synteny: Evolutionary Rearrangements, Plasticity, Human Disease Genes and Cancer Breakpoints

**DOI:** 10.3390/genes11040383

**Published:** 2020-04-01

**Authors:** Rita Scardino, Vanessa Milioto, Anastasia A. Proskuryakova, Natalia A. Serdyukova, Polina L. Perelman, Francesca Dumas

**Affiliations:** 1 Department of Biological, Chemical and Pharmaceutical Sciences and Technologies (STEBICEF), University of Palermo, 90100 Palermo, Italy; rituccia1989@gmail.com (R.S.); vanessa.milioto@unipa.it (V.M.); 2 Institute of Molecular and Cellular Biology, SB RAS, Novosibirsk 630090, Russia; andrena@mcb.nsc.ru (A.A.P.); serd@mcb.nsc.ru (N.A.S.); polina.perelman@gmail.com (P.L.P.)

**Keywords:** FISH, evolution, human synteny, painting, BAC probes, plasticity

## Abstract

The history of each human chromosome can be studied through comparative cytogenetic approaches in mammals which permit the identification of human chromosomal homologies and rearrangements between species. Comparative banding, chromosome painting, Bacterial Artificial Chromosome (BAC) mapping and genome data permit researchers to formulate hypotheses about ancestral chromosome forms. Human chromosome 13 has been previously shown to be conserved as a single syntenic element in the Ancestral Primate Karyotype; in this context, in order to study and verify the conservation of primate chromosomes homologous to human chromosome 13, we mapped a selected set of BAC probes in three platyrrhine species, characterised by a high level of rearrangements, using fluorescence in situ hybridisation (FISH). Our mapping data on *Saguinus oedipus, Callithrix argentata* and *Alouatta belzebul* provide insight into synteny of human chromosome 13 evolution in a comparative perspective among primate species, showing rearrangements across taxa. Furthermore, in a wider perspective, we have revised previous cytogenomic literature data on chromosome 13 evolution in eutherian mammals, showing a complex origin of the eutherian mammal ancestral karyotype which has still not been completely clarified. Moreover, we analysed biomedical aspects (the OMIM and Mitelman databases) regarding human chromosome 13, showing that this autosome is characterised by a certain level of plasticity that has been implicated in many human cancers and diseases.

## 1. Introduction

Comparative chromosome banding, followed by the advent of mapping by fluorescence in situ hybridisation (FISH), whole Chromosome Painting (CP) and Bacterial Artificial Chromosome (BAC) probes, have been used to detect chromosomal homologies, rearrangements and breakpoints among many mammalian species, defining major pathways of chromosome evolution in the class [1,2,3,4,5,6]. Indeed, these data are then analysed using cladistics and parsimony in order to define ancestral chromosomal syntenies as well as derived ones [2]. All of these approaches have paved the way to a reconstruction of the evolutionary history of human (*Homo sapiens*, HSA) chromosomes. 

The CP approach consists in the localisation of a whole chromosome probe mapped onto cytogenetic preparations by FISH [2,7]. First, human chromosome probes are mapped onto metaphases of target species; then animal probes of the target species can be mapped in a reciprocal hybridisation (RP) [8] on human metaphases, or on other animal genomes in an approach known as Zoo-FISH (Z-F) [9]. The analysis of these data regarding a single chromosome permits researchers to track each inter-chromosomal change involving the human chromosome under study. In yet another approach, human DNA sequences cloned inside vectors such as BACs are used as a mapping probe hybridised onto metaphases of target species, at a finer level, permitting the detection of fine chromosomal dynamics; these latter chromosome changes consist of small intra and inter-chromosomal rearrangements such as inversions, Evolutionary New Centromeres (ENC; new centromeres arise without the occurrence of inversions, maintaining the marker order) and duplications [3] which are not detectable by painting [5,6]. Animal BACs can be purchased from de Jong P. at the BAC/PAC Resource Centre (BPRC). Many works regarding the reconstruction of human chromosomes have been published so far, mainly by mapping BAC probes onto primate chromosomes [2,10].

Other chromosome features can be evaluated by mapping specific loci and repetitive probes that permit the localisation of sequences often believed to be responsible for the plasticity of chromosomes [11,12,13,14,15,16,17] and the detection of human genes involved in cancers [18].

Currently, homologies can be analysed by the Sequence Alignment (SA) data integrated with cytogenetic information using the new approach known as cytogenomics [19,20,21,22,23]. Sequence data are available from genomic browsers such as NCBI (https://www.ncbi.nlm.nih.gov/), UCSC (https://genome.ucsc.edu/) and Ensembl (https://www.ensembl.org/index.html), and can be integrated with molecular cytogenetic ones to analyse chromosome features, phylogenomic hypotheses and chromosome organisation.

All these approaches are useful not only to analyse chromosomal rearrangements that affect the syntenies, to define real homologies and the order of homologous sequences to avoid false breakpoints, but also to identify segmental duplications and copy number variants. Indeed, determining the order of conserved chromosome segments in the genomes of mammals is important not only for phylogenetic purposes but also for understanding speciation events and lineage specific adaptations [23].

The human chromosome 13 (HSA13) sequence has been released [24]; it is the largest acrocentric chromosome (114.36MB) in the karyotype, with 1381 genes, 41 novel genes and 477 pseudogenes; furthermore, it is among the human chromosomes with the lowest percentage of duplicated sequences [25]. Classic and molecular cytogenetic approaches (banding, CP and BAC mapping) allow researchers to formulate hypothesis about its evolutionary conservation despite some fusion or fission events in a few taxonomic groups [2,26,27]. The analysis of BAC probe signals on chromosomes of representative mammalian species permits researchers to make an initial reconstruction of the history of the chromosome, but particular focus has been given to the potential relationships between ENCs and neocentromeres occurring in clinical cases [27], as indeed has been previously shown [28]. Furthermore, a revision on human synteny 13 evolution in eutherian mammals has been recently proposed in a comprehensive review [29]. Furthermore, the cytogenomic approach applied by Kim and colleagues [23] showed that, in general, human chromosome evolution has been affected mostly by inversions and complex rearrangements observed during the evolution of the eutherian ancestor to human, whereas fusions and fissions were less prevalent. In this perspective, in order to study and verify the conservation of syntenic homologues to human chromosome 13 and to look for intrachromosomal rearrangements not easily detectable through other methods, we used FISH to map specific BAC probes in platyrrhine species. The species were chosen among taxa characterised by a high level of rearrangements, which suggested that they could be a useful model for the study of chromosome evolution. Our mapping data on *Saguinus oedipus* (Linnaeus, 1758), *Callithrix argentata* (Linnaeus, 1766), also known as *Mico argentata,* and *Alouatta belzebul* (Linnaeus, 1766) let us discuss chromosome 13 evolution in light of previously published data, with a comparative perspective involving not only primates. Indeed, we also made an update regarding cytogenomic literature data on human chromosome 13 evolution in eutherian mammals [29], permitting us to reconstruct the main evolutionary steps of human 13 synteny. Furthermore, we analysed the OMIM and the Mitelman databases on human chromosome 13 in order to shed light on its plasticity and other evolutionary and biomedical aspects.

## 2. Materials and Methods

Following the standard protocol [30], metaphases were obtained for specimens of cotton-headed tamarin, *Callithrix argentata (CAR)*, silvery marmoset, *Saguinus oedipus (SOE)* (Cebidae) and red-handed howler, *Alouatta belzebul (ABE)* (Atelidae) from primary fibroblast cell lines or lymphoblasts (human), detailed in Table 1.

All experiments were performed in accordance with relevant institutional and national ethical guidelines and regulations. 

Samples were karyotyped by G-banding or DAPI inverted banding. BAC probes for human chromosome 13 were purchased from de Jong P. J. of the BACPAC Resources Center (BPRC), Oklahoma, USA (currently in Richmond, CA, USA). The BACs with human chromosome 13 sequences from the RP11 library were selected from the UCSC genome browser (GRCh 37/Dec 2004). *Escherichia coli* (Migula, 1895) with human chromosome 13 sequences cloned in BACs were grown in LB broth (Gibco, ThermoFisher Scientific, MA, USA). BAC DNA was isolated by BIORAD Miniprep Kit (CA, USA), and amplified by a Whole Genome Amplification Kit, WGA1 (Sigma Aldrich, St. Louis, MO, USA). BAC probe labelling was performed by adding dUTP with fluorochromes using a WGA3 Kit (Sigma Aldrich, St. Louis, MO, USA).

In situ hybridisation of probes on the chromosomal spreads was performed according to previously published protocols [10] using an avidin-FITC/biotinylated anti-avidin system or DIG/Alexa system (Invitrogen, ThermoFisher scientific). Probes were also mapped on human metaphases as control.

BAC signals, for each species studied, were analysed by microscope Axioscope 2 (ZEISS, Jena, Germany). Chromosomes with BAC signals were identified by inverted DAPI or G-banding in accordance with previous painting data.

Furthermore, previous chromosome painting and genome data on human chromosome 13 evolution were analysed and upgraded presenting data on the mammalian molecular phylogenetic tree, drawn according to previous reconstructions [23,29,31] and modified using the Mesquite program v.2.75 [32]. The platyrrhine tree was also here drawn using the same program and according to a previous molecular phylogenetic reconstruction [4].

We analysed disease loci (365) reported for HSA13 chromosome in the OMIM database (http://www.ncbi.nlm.nih.gov/Omim/mimstats.html) as well as many cancer breakpoints (519) involving this chromosome from the Mitelman database (http://www.gap.nci.nih.gov.Chromosomes/Mitelman) in order to study the distribution of the disease loci, considering only representative ones with phenotype (109 among 365) and all cancer breakpoints.

## 3. Results

**Figure 1:**
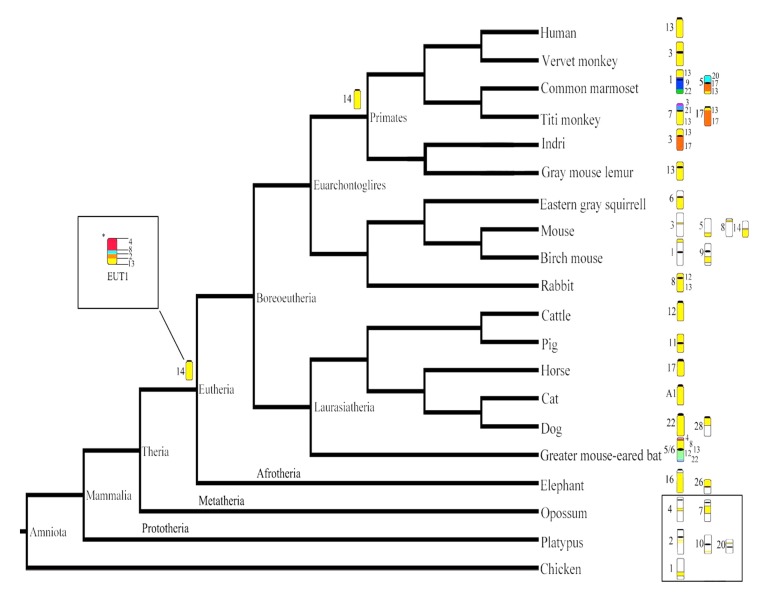
The mammalian phylogenetic tree show the orthologous blocks that correspond to human chromosome 13 (in yellow) in representative eutherian species for which reciprocal painting data is available; the tree presents data from Table 2. Chromosome ideograms on which human synteny 13 is found are reported for each species, and the species’ chromosome number is shown on the left of the ideograms and HSA syntenies on the right. When HSA13 synteny, yellow, is rearranged with just a few human syntenies, these are represented in different colours and are reported on the right of the ideogram (for example, in indri, chromosome 3, synteny 13 (yellow), is fused with synteny 17 (red)), while when HSA 13 (yellow) is rearranged with many other human syntenies, these are represented by white segments for logistic reasons (for example, on chicken chromosome 1). For some species, DNA sequence alignments have been done previously, see Table 2 for citations. On the tree, the ancestral synteny 13 form (chromosome 14) described by painting data analysis is reported, and the eutherian ancestral chromosome 13 (EUT 1) alternative reconstruction, obtained through sequence data, from Table 2, is shown in the upon box on the right of the tree underlined with an asterisk * [23]. Platypus (Monotremata), opossum (Marsupialia) and chicken (Aves) chromosome homologues are reported in the box at the lower right; these species are representative outgroups. Synteny homologues to human chromosome 13 are on chromosomes 2, 10 and 20 in the platypus, chromosomes 4 and 7 in the opossum and chromosome 1 in the chicken. Black areas indicate the centromere. The tree topology constructed according to previous data [31], and also considering results from [23] was modified from Scardino et al. [29]

The literature data on human chromosome 13 evolution, here considered in Table 2 accordingly with [29], were analysed and presented on the mammalian molecular phylogenetic tree [23,29,31]. This tree was drawn and modified using the Mesquite program v.2.75 [32] (Figure 1).

**Figure 2:**
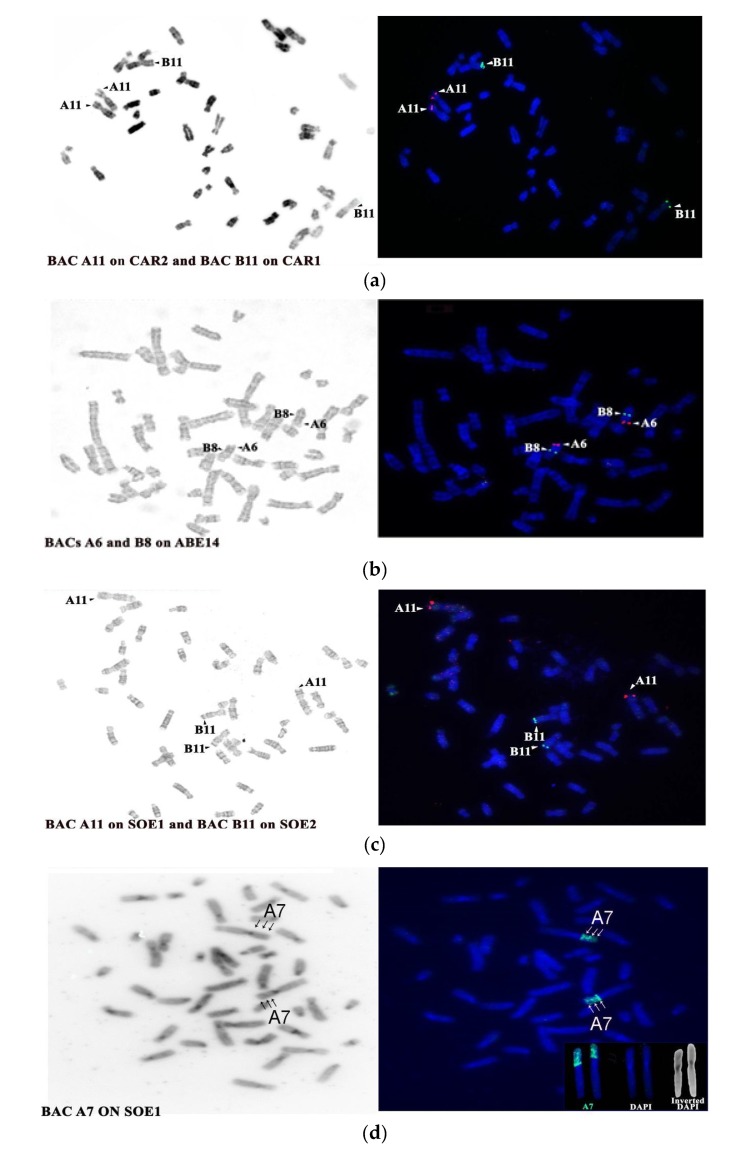
Examples of fluorescent in situ hybridisation of mapped BACs onto CAR (**a**), ABE (**b**), SOE (**c**,**d**) metaphases. Note that BAC A7 on SOE chr 1p (**d**) shows triple band signals.

Bright signals of the BAC probes on the chromosomes of the analysed species are shown (Figure 2); human synteny 13 homologues were two chromosomes in both *Saguinus oedipus* (chr 1, 2) and *Callithrix argentata* (chr 1, 2) and a single synteny in *Alouatta belzebul* (chr 14), in accordance with previous chromosome painting data [44,49]; these chromosomes have been identified using inverted DAPI banding or G-banding.

BAC types and mapping results are summarised in Table 3.

**Figure 3:**
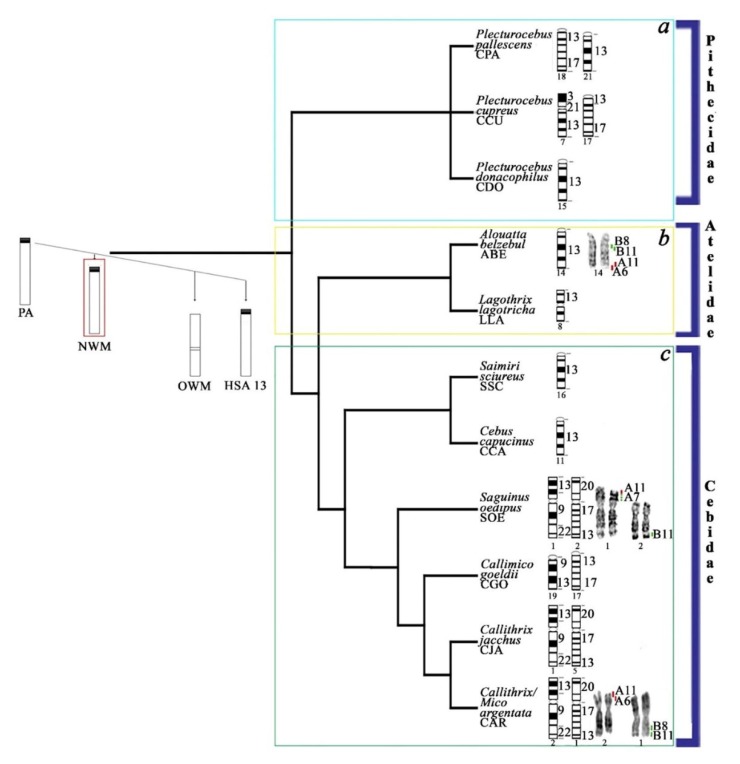
The platyrrhine phylogenetic tree showing the ideograms of the chromosome homologous to HSA13 synteny in representative New World monkeys (NWM). BAC probe mapping (red and green signals) are reported on the right side of banded chromosomes. The species’ chromosome numbers are reported under the ideograms, and HSA synteny associations are on the right. Note that some species previously recognised among the *Callicebus* genus have now been placed among the new *Plecturocebus* genus in recent molecular phylogeny [103]. PA—primate ancestor, OWM—Old World monkeys. The tree topology has been here reconstructed and modified according to previous analyses and reconstructions [4,104].

These results were compared with other species previously analysed using the same approach [27]. The painting data (Table 2) and BAC mapping signals on synteny HSA13 are reported on the platyrrhine phylogenetic tree (Figure 3), here drawn in accordance with a previous reconstruction [4], but with some modifications using the Mesquite program v.2.75 [32].

**Figure 4:**
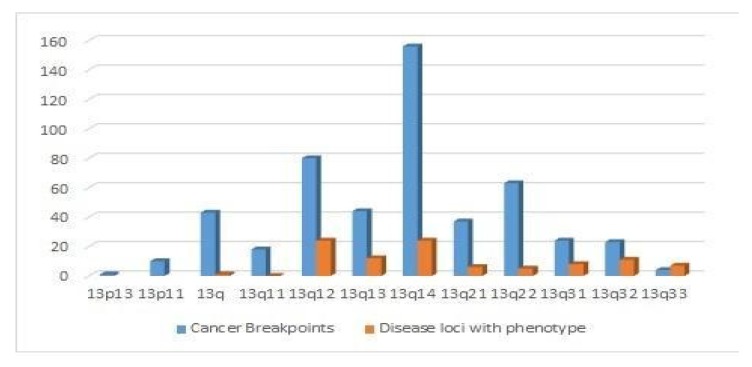
Distribution by band (x-axis) of 109 disease loci (orange) and 519 cancer breakpoints (blue), described in the OMIM and Mitelman databases, respectively (Tables S1 and S2). (Note that q12–q14 bands are particularly involved, not just in medical aspects but also in inter-chromosomal rearrangements occurring during evolution, as in Platyrrhini).

Furthermore, we analysed disease loci (365) reported for HSA13 in the OMIM database and many cancer breakpoints (519) involving this chromosome from the Mitelman database. The analysis permitted us to construct a histogram to study the distribution of the disease loci (Figure 4) considering only representative ones with phenotype (109 among 365) (Table S1), and all cancer breakpoints ordered on chromosomal bands along human chromosome 13 (Table S2).

## 4. Discussion

In this work, we have delineated the main steps regarding the evolutionary history of human chromosome 13 synteny in mammals. This chromosome history was traced based on comparative chromosome painting and mapping data. Furthermore, we performed comparative cytogenomic analysis and BAC mapping by FISH, with particular attention to platyrrhine species (Primates). These latter data were compared with those for other mammal species, in particular other primates, which had been previously analysed using the same approaches.

We also show chromosome 13 implicated in many human tumour formations and diseases; furthermore, we show that studies of chromosomes in an evolutionary perspective could shed light on the pattern of changes in correlation with cancer breakpoints or peculiar sequences that may be responsible for disease occurrence. In particular, we show that the region chr13q12–q14 is involved in both evolutionary changes and disease events.

### 4.1. Evolutionary History of HSA13 Synteny in Eutherian Mammals

The main steps of the evolution of human chromosome 13 synteny have been reconstructed and upgraded, considering previous molecular cytogenetic data obtained by painting and sequence analysis (Table 2), [29]; the steps are illustrated in a graphical reconstruction of the mammalian phylogenetic tree (Figure 1); this mammalian phylogenetic tree reconstructed in agreement with previous ones [23,29,31], has been drawn using Mesquite 2.75 [32].

Mammals are categorised into three major groups: monotremes (Prototheria, platypus), marsupials (Metatheria, opossum) and placental mammals (Eutheria), with these last two known as Theria; among placental mammals, Afrotheria, Xenarthra and Boreoeutheria are recognised, with the latter comprising Laurasiatheria and Euarchontoglires (or Supraprimates) [31]. Synteny 13 orthologues are reported in the mammalian phylogenetic tree for representative eutherian species for which RP is available; molecular sequence alignments are also reported for some of them, when available (Table 2). Chromosome painting data let us propose that synteny 13 is conserved in mammalian orders as a single chromosome, as seen in Dermoptera, Pilosa, Carnivora, Perissodactyla and Cetartiodactyla but with exceptions in each group; for example, in cow—*Bos taurus*, chr 12 and pig—*Sus scrofa*, chr 11; in pigs, however, the synteny is on the metacentric chromosome due to the formation of a new centromere. Many rearrangements can be seen in other groups: Afrotheria (Tubulidentata, Afrosoricida, Macroscelidea, Sirenia), Eulipotyphla, Pholidota, Chiroptera, Rodentia where synteny 13 is associated with one or more human syntenies due to translocation; for example, among Chiroptera, in the greater mouse-eared bat *Myotis myotis*, human synteny 13 is present on chromosome 5/6, associated with many other human syntenies (8/4/13/12/22), and among Rodentia, in the eastern grey squirrel *Sciurus carolinensis*, human synteny 13 is present on chromosome 6, associated with other human syntenies (reported in white in Figure 1) and in Lagomorpha with the rabbit—*Oryctolagus cuniculus*, chr 8 covered by the human association 12/13. Furthermore, human synteny 13 can be fragmented into two or more segments and associated with other HSA syntenies; for example, in carnivores (dog—*Canis familiaris*, chr 22, 28), Proboscidea (elephant—*Loxodonta africana*, chr 16, 26) and Rodentia species such as the birch mouse (*Sicista betulina*, chr 1, 9).

Analysing sequence alignments available from genomic browsers, chromosome 13 homologues are conserved in many mammals such as pigs, horses and cats [20], in agreement with painting data, and it is very fragmented in mice (*Mus musculus*, chr 3, 5, 8, 14) [21]; however, in outgroups such as the opossum (*Monodelphis domestica*, ch 4, 7), the chicken (*Gallus gallus*, chr 1) [101,102] and the platypus (*Ornithorhynchus anatinus*, chr 2, 10, 20) [100], through SA it has also been shown to be fragmented (Table 2, Figure 1). Indeed, recently, the analysis of many placental mammals has allowed researchers to hypothesise that the eutherian ancestral chromosome HSA13 was fused with other human syntenies (HSA4, and parts of HSA 2 and 8) [23]; in the latter reconstruction in Figure 1, synteny 13 on eutherian mammals (EUT) chromosome 1 is associated with other HSA syntenies (HSA4/8/2/13) and is in agreement with previous SA data [23]. Our chromosome painting analysis shows, on the other hand, these kind of human associations (13/2/8/4) involving human synteny 13 only on the greater mouse-eared bat’s chromosome 5/6, (HSA 4/8/13/12/22) [89]; thus, the molecular reconstruction of this ancestral form does not find support through painting. Consequently, the two reconstructions of ancestral synteny 13 in eutherians, by painting and sequence analysis, are not consistent. To better clarify this complex origin would require using appropriate outgroups and filling the gap in the incomplete set of taxa analysed so far. In particular, the lack of comparative chromosome painting between eutherians, monotremes and marsupials, and the lack of genomic data, do not permit a better reconstruction [23,29,98].

At a finer level, through BAC mapping applied to some representative mammals, common small intrachromosomal rearrangements have been shown along human chromosome 13 homologues in non-primate mammals [27], in agreement with SA data [23]. Moreover, evolutionarily new centromeres potentially linkable to neocentromeres are common, such as those shown in pig and *Lagothrix lagothricha* chr 8 (Platyrrhini).

### 4.2. Evolutionary History of HSA 13 Synteny in Primates

The first reconstruction of human chromosome 13 synteny was proposed using classical cytogenetics applied to Anthropoidea [1,26,105]; it is an acrocentric chromosome in great apes, with a paracentric inversion only in *Gorilla gorilla* [105]. In prosimians, the human chromosome 13 homologue is present as a single conserved synteny, but possibly metacentric, presumably due to an inversion or, alternatively, to the presence of a new centromere, as in *Microcebus murinus* (chromosome 13). On the other hand, it could be rearranged, as in *Indri indri* (chromosome 3), where it is associated with human synteny 17 (Figure 1). In catarrhines, a new centromere changed morphology from acrocentric to metacentric, as in *Chlorocebus aethiops*. While it is relatively conserved in these species, it is much more rearranged in platyrrhines.

### 4.3. Evolutionary History of HSA13 Synteny in Platyrrhines

The main evolutionary steps indicated by chromosome painting data on Platyrrhini regarding synteny HSA 13 (Table 2) are illustrated in a graphical reconstruction of the platyrrhine phylogenetic tree, drawn here using Mesquite (Figure 3). Even though human chromosome 13 is presumably conserved in the ancestors of platyrrhines, HSA 13 homologues have undergone many rearrangements in New World monkeys. Among platyrrhines, three families are recognised: Pitheciidae, Atelidae and Cebidae (Figure 3). Among Pitheciidae (Figure 3a) in *Callicebus* (also known as *Plecturocebus* [103]), we found conservation of synteny HSA 13, as demonstrated in *C./P. donacophilus*, chromosome 15 [60], while it is split in some others species, for example, *C./P. pallescens*, on chromosomes 18 and 21 [61]. Furthermore, on chromosome 18, synteny HSA 13 is associated with human synteny 17. In this latter species, breakpoints have also been evaluated, occurring in position q12.3 [61]. A similar organisation is found in Titi monkeys (*C./P. cupreus*) on chromosomes 7 and 17 (covered, respectively, by HSA 3/21/13 and 13/17 [61]). Among Atelidae (Figure 3b), synteny 13 appears to be conserved, as in *Alouatta belzebul* chromosome 14 [57] and *L. lagothricha* chromosome 8 [27]; however, in this latter species, the chromosome is metacentric and a new centromere has been shown by BAC probe mapping [27]. Among Cebidae (Figure 3c), synteny 13 is conserved in a single chromosome, for example, on *Cebus capucinus* chromosome 11 [52] and *Saimiri sciureus* chromosome 16 [50], or fissioned, with subsequent translocation to other HSA syntenies. This latter arrangement is found, for example, in common marmosets (*Callithrix jacchus*), resulting in the formation of chromosomes 1 and 5 (covered respectively by HSA 13/9/22and 13/17/20, Figure 1a) [44]. In addition, in *Callimico goeldii*, synteny 13 is fragmented into two segments forming chromosomes 17 and 19, with chromosome 17 covered by the syntenic association 13/17 and chromosome 19 by synteny 13 with little parts of synteny 9 [8,44].

In the species analysed in the present work, human synteny 13 homologues have also been split into two fragments on chromosomes 1 and 2 of both SOE and CAR (Cebidae); on SOE, the breakpoint has been evaluated by RP in position 13q13 [8,44]. On the metacentric SOE 1 and CAR 2, human synteny 13 is associated with human synteny 9 (13/9/22), and on chromosomes SOE 2 and CAR 1, synteny 13 is associated with human synteny 17 (20/17/13); this organisation is the same as that found in *Callithrix jacchus* chromosome 1 and 5. The mapping of our BAC probes was in agreement with the painting data (Figure 3c), in particular, probe A6/A11 on the terminal position of p arm of chromosome SOE 1 and CAR 2, and probes B8 and B11 on SOE 2 and CAR 1 on the q arm in a terminal position (Figure 2 and Figure 3c). In ABE (Atelidae), all four probes hybridised onto chromosome 14, with B8 and B11 interstitial underneath the black band below the centromere and A11 and A6 at the terminal position; this same position was found for the probes that were applied onto HSA metaphases as control. Those data have been compared with the BAC probes of the same coordinates applied in a previous work on other platyrrhine species, such as *Callithrix jacchus*, *Saimiri sciureus* (Cebidae), *L. lagothrica* (Atelidae) and *Callicebus moloch* (Pithecidae) [27]. Human association data reported in the latter work on *Callithrix jacchus* needed to be revised due to the fact that synteny 13 is fissioned into two fragments on CJA chromosomes 1 and 5, which are covered, respectively, by human syntenic associations 13/9/22 and 20/17/13, in agreement with the painting data [44]; moreover, *Callicebus moloch* should instead be considered as *C. pallescens* due to the fact that this latter species’ synteny is fissioned into two fragments: one covering chromosome 21 and one on chromosome 18, where it is in association with HSA synteny 17 [61], as correctly reported in Figure 3c for further comparisons. 

Our analysis showed marker order conservation of the BAC probes considered in all species compared, even where synteny 13 had been split during evolution; but we found an exception for probe A7 that had been mapped on SOE, where it showed three mapping signals on chromosome 1 (Figure 2 and Figure 3c), in agreement with previous BAC mapping in *Callithrix jacchus* chromosome 1p [27]. These data indicate that a complex rearrangement in SOE chromosome 1p occurred, probably an inversion or duplication. This small rearrangement, demonstrable only through BAC mapping, is also present on CJA chromosome 1 as it possible to see when the same probe position is considered [27]. Further analysis is needed to show whether the small and not easily detectable rearrangement is shared with others Cebidae. 

Our analysis of the organisation of synteny 13 in platyrrhine species shows that it is not as conserved as previously shown. Indeed, in the three families, we show both ancestral and derived organisation of human synteny 13. The derived form of synteny 13 often has breakpoints occurring between q12.2 and q14. Furthermore, probe mapping shows marker order conservation with exceptions. In light of these results, better analyses at a finer level are needed in order to better understand the evolution of synteny 13 and to help define homologies useful for phylogenetic and adaptive interpretation [23].

### 4.4. Distribution of Cancer Breakpoints and Loci Implicated in Human Diseases

The distribution of the loci considered, representative ones with phenotype (109 among 365) (Table S1), and all cancer breakpoints ordered by chromosomal bands along human chromosome 13 (Table S2), does not appear to be uniform. Indeed, there are some bands (13q12 and 13q14) that are clearly more affected than others, as it is possible to observe in the frequency histograms we have produced (Figure 4). These data are in agreement with evolutionary synteny 13 changes occurring in the same region in Platyrrhini. In general, it has been shown that the most frequent cancer-associated chromosomal aberrations affecting tumour genes are close (at less than 0.4 MB) to reuse breakpoint regions identified through multispecies genome comparison [20]. This evidence is in agreement with other frequent human cancer-associated breakpoints that are co-localised with evolutionary breakpoints more frequently than other, less common, neoplasms. The coincidence of frequently occurring cancer breakpoints with evolutionary breakpoints indicates that some of the features that induce fragility in certain evolutionary breakpoints in germ lines are still retained in the human genome [22]. On chromosome 13, the most frequent cancer-associated chromosomal aberration close to reuse breakpoints identified from multispecies genome comparisons are the ones involving: gene *ZNF198* (ZMYM2) translocation t(8;13) (p11;q12), which is linked to chronic myeloproliferative disorder and stem cell leukaemia lymphoma syndrome (SCLL); and gene *FOXO1* translocation t(2;13)(q36;q14), linked to alveolar rhabdomyosarcoma. Furthermore, many other disease loci have been described. Indeed, chromosome 13 contains genes involved in other chromosomal aberrations, such as those involving the *LCP1* gene translocation t(3;13)(q27;q14), linked to follicular lymphoma, *BCL6* translocation t(3;13)(q27;q14), linked to B-cell non-Hodgkin lymphomas (B-cell NHL). Moreover, it is rearranged with the deletion del(13)(q13q14) in B-cell chronic lymphocytic leukaemia (CLL), the translocations t(12;13)(p13;q12) and t(12;15)(13p;q25) are common in acute myeloid leukaemia (AML), translocation t(12;13)(p13;q14) in acute lymphoblastic leukaemia (ALL) and deletion del(13)(q13q14) in retinoblastoma (*RB1*). Chromosome 13 is also responsible for type 2 breast cancer (*BRCA2*) and contains the *DAOA* locus associated with bipolar disorder and schizophrenia, among many other genetic diseases (Table S1). Since it has been shown that some genetic diseases or cancer breakpoints occur in correspondence with the same peculiar region along the chromosomal arms linked to the presence of repeated sequences or segmental duplications [25], or in correspondence with reuse breakpoints which arose during evolution [20,22], the study of the distribution of the loci along the chromosome and the features that could induce fragility in the sequence would be a promising path to pursue.

## 5. Conclusions

Our analysis of human chromosome 13 synteny, updating a previous data report [29], permits us to define conserved segments but also to show that it is less conserved than previously thought. In particular, we have reviewed previous data regarding inter-chromosomal rearrangements detectable through painting, and intra-chromosomal rearrangements detected by BAC mapping, on synteny HSA 13. We have focused our attention on primates, in particular on platyrrhines, where we show a highly inter-chromosomal and intra-chromosomal dynamic. Furthermore, we show chromosome 13 as an autosome that is commonly implicated in human tumour formation and diseases; the study of chromosomes in an evolutionary perspective shed light on the pattern of changes in correlation with cancer breakpoints or particular sequences that may be responsible for disease occurrence. In particular, we show that the region between q12–q14 is involved both in evolutionary changes and disease events, and for this reason it deserves special attention.

## Figures and Tables

**Table 1:** List of cell lines used in this study.

Family	Latin Name	Code	Cell Type	Sample/Cell Line Acknowledgement
Cebidae	*Saguinus oedipus*	SOE	fibroblast cell line	Melody Roelke (Frederick National Laboratory of Cancer Research, Leidos Biomedical Research, Frederick, MD, USA), June Bellizzi and Director Richard Hann (Catoctin Wildlife Park and Zoo, Thumont, MD, USA)
Cebidae	*Callithrix argentata*	CAR	fibroblast cell line	Stephen O’Brien (Laboratory of Genomic Diversity, National Cancer Institute, Frederick, MD, USA) and Hector Seuánez (Departamento de Genética, Instituto de Biologia, Universidade Federal do Rio de Janeiro, Brazil)
Atelidae	*Alouatta belzebul*	ABE	fibroblast cell line	

**Table 2:** The list of species analysed by Chromosome Painting (CP), Reciprocal Painting (RP), Zoo-FISH (Z-F) and/or comparison of Sequence Alignments (SA) and respective references. For each species, human chromosome 13 homologues and other human associations are reported. The * indicates an alternative chromosome identification reported in a different reference. The shaded areas in the second column indicate ancestral conserved state of human chromosome 13 (HSA13) synteny as a single chromosome element: green shading—acrocentric morphology, light green shading—non-acrocentric morphology, prox—proximal part, ter—terminal part, q—q-arm, p—p-arm. Note that this table is a modified version of the original by Scardino et al. [29].

Species	Chromosome Morphology	Chr. Num.	Human Chromosome Association	Reference	Method
**MAMMALIA**					
EUTHERIA					
BOREOEUTHERIA					
EUARCHONTOGLIRES					
**PRIMATES**					
**Catarrhini**					
*Homo sapiens*	**A**	**13**			
*Pan troglodytes*	A	14		[7]	CP
*Gorilla gorilla*	A	14		[7]	CP
*Pongo pygmaeus*	A	14		[7]	CP
*Hylobates concolor*	M	5, 9	1/13; 1/4/10/13	[33]	CP
*Hylobates klossii*		4q	3/13	[34]	CP
*Hylobates moloch*		4q	3/13	[34]	CP
*Hylobates lar*		4q	3/13	[7]	CP
*Symphalangus syndactylus*	M	15		[7]	CP
*Pygathrix nemaeus*	SM	17		[35]	CP
*Nasalis larvatus*	M	15		[36]	CP
*Semnopithecus francoisi*	M	9		[37]	CP
*Semnopithecus phayrei*	M	9		[34]	CP
*Presbytis cristata*	M	19		[38]	CP
*Colobus guereza*	M	19		[39]	CP
*Erythrocebus patas*	SM	15		[40]	RP
*Chlorocebus aethiops*	M	3		[41]	CP
*Cercopithecus erythrogaster*	SM	12		[42]	Z-F
*Cercopithecus stampflii*	SM	13		[42]	Z-F
*Cercopithecus neglectus*	M	19		[40]	RP
*Macaca fuscata*	SM	16		[43]	CP
**Platyrrhini**					
*Cebuella pygmaea*	SMs	1, 4	13/9/22, 20/17/13	[44] [8]	CP RP
*Callithrix argentata*	SMs	2, 1	13/9/22, 20/17/13		
*Callithrix jacchus*	SMs	1, 5	13/9/22, 20/17/13		
*Callimico goeldii*	As	19, 17	13/9/22, 13/17		
*Saguinus oedipus*	SMs	1, 2	9/13/22, 20/17/13		
*Leontopithecus chrysomelas*	SMs	1, 2	9/13/22,13/17/20	[45]	CP
*Aotus nancymaae*	A	19		[46]	CP
*Aotus infulatus*	A	14		[47]	CP
*Aotus lemurinus griseimembra*	A	17		[46,48,49]	CP
*Saimiri sciureus*	A	16		[8,50]	CP
*Cebus (Sapajus) apella*	A	17		[51,52]	CP, Z-F
*Sapajus a. paraguayanus*	A	17		[52]	Z-F, CP
*Sapajus a. robustus*	A	17			
*Cebus capucinus*	A	11			
*Cebus nigrivitatus*	A	17		[53]	CP
*Lagothrix lagotricha*	SM	8		[54]	CP
*Brachyteles arachnoides*	A	20		[55]	CP
*Ateles paniscus paniscus*	M	4	13a/13b/3c/7b/1a2		
*Ateles belzebuth marginatus*	SM	12			
*Ateles geoffroyi*	SM	12		[56]	CP
*Ateles belzebuth hybridus*	A	12		[53]	CP
*Alouatta belzebul*	A	14		[57]	CP
*Alouatta seniculus sara*		12		[57]	CP
*Alouatta seniculus arctoidea*		16		[57]	CP
*Alouatta caraya*	A	15 (20 *)		(* [48]) [49,58]	CP
*Alouatta seniculus macconnelli*	SM	4q	13/19	[58]	CP
*Alouatta guariba guariba*	A	14		[48,49]	CP
*Cacajao calvus rubicundus*	A	13		[59]	CP
*Chiropotes israelita*	A	15		[46]	CP
*Chiropotes utahicki*	A	15		[46]	CP
*Pithecia irrorata*	SM	8	22/13	[59]	CP
*Plecturocebus (Callicebus) donacophilus pallescens*	A	15		[60]	CP
*Plecturocebus (Callicebus) cupreus*	SMA	7, 17	3/21/13, 13/17	[61]	CP
*Plecturocebus (Callicebus) pallescens*	A	18, 21	13/17, 13	[61]	CP,
*Cheracebus (Callicebus) lugens*	SM	1	1/13 - 12/13	[62]	CP
*Callicebus moloch*	A	21		[50]	CP
*Callicebus personatus*	M	1	13/20	[63]	CP
*Callicebus nigrifrons*	A	4, 17	13/20, 13/17	[64]	CP
**Strepsirrhini**					
*Lemur catta*	A	13		[65] [66]	BAC CP
*Hapalemur griseus griseus*		15		[66]	CP
*Eulemur fulvus*	A	12		[66]	CP
*Microcebus murinus*	SM	13		[67] [66]	CP
*Lepilemur edwardsi*		6p		[67]	CP
*Lepilemur ankaranensis*		14		[67]	CP
*Lepilemur jamesi*		5q ter		[67]	CP
*Lepilemur leucopus*		1q ter		[67]	CP
*Lepilemur microdon*		5p		[67]	CP
*Lepilemur mittermeieri*		7p		[67]	CP
*Lepilemur dorsalis*		6p		[66,67]	CP
*Lepilemur mustelinus*		8 ter		[66,67]	CP
*Lepilemur ruficaudatus*		5q prox		[66,67]	CP
*Lepilemur septentrionalis*		14		[67] [66]	CP
*Lepilemur dorsalis*		6p		[67] [66]	CP
*Lepilemur mustelinus*		8 ter		[67] [66]	CP
*Lepilemur ruficaudatus*		5q prox		[67] [66]	CP
*Lepilemur septentrionalis*		14		[67] [66]	CP
*Indri indri*	SM	3p	13/17	[66]	CP
*Propithecus verreauxi*		6q	5/13	[66]	CP
*Avahi laniger*		12		[66]	CP
*Daubentonia madagascariensis*		8p	10/13	[66]	CP
*Nycticebus coucang*	SM	18 17		[68] [69]	RP CP
*Galago moholi*	M	5	13/16/12	[70]	CP
*Otolemur garnettii*	SM	14		[68]	RP
*Otolemur crassicaudatus*	A	14		[70]	CP
**DERMOPTERA**					
*Galeopterus variegatus*	A	13		[71]	RP
**SCANDENTIA**					
*Tupaia belangeri*	A	17		[72]	CP
*Tupaia minor*	A	16		[73]	CP
**LAGOMORPHA**					
*Oryctolagus cuniculus*	SM	8	13/12	[74] [75]	RP SA
**RODENTIA**					
*Mus musculus*		3, 5, 8, 14, 14		[20,21] [23,75]	SA
*Rattus norvegicus*		2, 12, 15, 15, 16		[20] [23]	SA
*Pedetes capensis*	SM	6	13/12/22	[76]	CP
*Sicista betulina*	M, SM	1, 9	13/4/10/11/9/10, 3/6/313/19	[76]	CP
*Castor fiber*	SM	4	8/13	[76]	CP
*Sciurus carolinensis*	SM	6	10/13	[77,78]	RP
*Petaurista albiventer*	M	11	10/13	[78]	CP
*Tamias sibiricus*	M	10	10/13	[78]	CP
LAURASIATHERIA					
**PHOLIDOTA**					
*Manis javanica*	SM M	1, 9q	13/5/2p, 18/13	[79] [80]	CP CP
*Manis pentadactyla*	SM A	1q, 17	13/5/2, 13	[80]	CP
**CARNIVORA**					
*Canis familiaris*	As	(25 *) 22, 28		(*[81]), [82] [75,83] [23]	RP CP Z-F SA
*Vulpes vulpes*	SMs	6, 9	13/14, 2/8/13/3/19	[82]	RP
*Mustela putorius*	SM	8		[84]	CP
*Procyon lotor*	M	3	13/2	[85]	CP
*Mephitis mephitis*	SM	19		[85]	CP
*Felis catus*		A1p	13/5	[82] [19,20], [75]	CP SA
**PERISSODACTYLA**					
*Tapirus indicus*	A	18		[9]	Z-F
*Diceros bicornis*	A	10		[9]	Z-F
*Ceratotherium simum*		10		[9]	Z-F
*Equus caballus*	A	17		[86] [20,23] [9] [27]	RP SA Z-F BAC
*Equus burchelli*	SM	6q	13/9	[9]	RP Z-F
*Equus asinus*		11		[9]	Z-F
*Equus grevyi*	SM	6q	13/9	[9]	Z-F
*Equus zebra hartmannae*		15		[9]	Z-F
*Equus hemionus onager*		5q	12/13/22	[9]	Z-F
*Equus przewalskii*		16		[9]	Z-F
**CETARTIODACTYLA**					
*Bos taurus*	A	12		[19] [23] [87]	SA RP
*Moschus moschiferus*	A	17		[88]	Z-F
*Okapia johnstoni*	A	11		[88]	Z-F
*Giraffa camelopardalis*	M	12	14/15/13	[88]	Z-F
*Globicephala melas*	M	15		[88]	Z-F
*Hippopotamus amphibious*	M	15		[88]	Z-F
*Sus scrofa*	M	11		[19] [23] [87]	SA RP
*Camelus dromedarius*	M	14		[87]	RP
**CHIROPTERA**					
*Mormopteurus planiceps*	M	M7	13/18	[89]	CP
*Myotis myotis*	M	V5/6	4/8/13/12/22	[89]	CP
*Taphozous melanopogon*	SM	1	4c/8b/13/16b/7c/5a	[90]	CP
*Megaderma spasma*	M	12	20/13/8b/4c	[90]	CP
*Rhinolophus mehelyi*	A	R6	13/4/8/13	[89]	CP
*Aselliscus stoliczkanus*	M	1	22/12/13/4/8/13	[91]	CP
*Hipposideros larvatus*	M	H1	13/3/21	[89] [91]	CP
*Eonycteris spelaea*	SM	E11	13/4/8/13	[89]	CP
**EULIPOTYPHLA**					
*Hemiechinus auritus*	SMs	5q, 6	5/13, 2/22/12/13/12	[79]	CP
*Neotetracus sinensis*	SM A	3, 10	13/4/20/10,1/13/10/12/22	[92]	CP
*Sorex araneus*	M	bc	9/5/2/13/8/7	[23,92]	CP, SA
*Blarinella griselda*	SM	3	13/10/13/4/5	[92]	CP
*Talpa europaea*	M	6	2/13	[93]	CP
ATLANTOGENATA					
AFROTHERIA					
**PROBOSCIDEA**					
*Loxodonta africana*	A, SM	16, 26	13, 6/13/3	[19,94] [23]	CP SA
*Elephas maximus*	A, SM	16, 26	13, 6/13/3	[94]	CP
**SIRENIA**					
*Trichechus manatus*	M	19	13/3	[95]	CP
**TUBULIDENTATA**					
*Orycteropus afer*	SM	1	19/16/13/2/8/4	[94] [96]	CP SA
**MACROSCELIDEA**					
*Elephantulus rupestris* *Elephantulus edwardii*	SM	2	13/3/21/5	[97] [96]	CP SA
*Macroscelides proboscideus*	SM	2	13/3/21/5	[98]	CP
**AFROSORICIDA**					
*Chrysochloris asiatica*	M	8	13/18	[97] [96]	RP SA
XENARTHRA					
**CINGULATA**					
*Dasypus novemcinctus*	SM	19		[99]	CP
**PILOSA**					
*Tamandua tetradactyla*	M	4, (2 *)	13/1	[79], (* [99])	CP
*Choloepus didactylus*	A	17		[79]	CP
*Choloepus hoffmanni*	A	12		[99]	CP
*Bradypus torquatus*	A	12		[100]	CP
*Bradypus variegatus*	A	17			
METATHERIA					
MARSUPIALIA					
**DIDELPHIMORPHIA**					
*Monodelphis domestica*	SMs	4, 7		[101,102]	SA
PROTOTHERIA					
**MONOTREMATA**					
*Ornithorhynchus anatinus*	SM Ms	2, 10, 20		[101]	SA
AVES					
**GALLIFORMES**					
*Gallus gallus*		1		[101,102]	SA

Chromosome morphology legend: Green–acrocentric, light green–submetacentric.

**Table 3:** The list of Bacterial Artificial Chromosome (BAC) clones used with their chromosome start coordinates and mapping position in the human genome from the UCSC genome browser (GRCH 37/Dec 2004) and the mapping position obtained on the three species analysed: *S. oedipus* (SOE), *C. argentata* (CAR), *A. belzebul* (ABE).

BAC Clone	Start Coordinates	Mapping Position			
		HSA	SOE	ABE	CAR
**A6** CHORI RP11-35m5	27534229	13 tel	1p tel/ three bands	14 tel	1p tel
**A7** CHORI RP11-85p8	27475788				
**A11** CHORI RP11-14a4	39027477	13 tel	1 p tel	14 tel	1p tel
**B8** CHORI RP11-30n18	48823331	13 cen	2q tel	4 interstitial below dark band	2q tel
**B11** CHORI RP11-54g17	44481779	13 cen	2q tel	14 interstitial below dark band	2q tel

## Supplementary Materials

The following are available online at https://www.mdpi.com/2073-4425/11/4/383/s1, Table S1: List of disease loci from the OMIM database, considering only representative ones with phenotype (109 among 365), ordered by chromosomal bands along human chromosome 13, Table S2: List of cancer breakpoints (519) from the Mitelman database, ordered by chromosomal bands along human chromosome 13.

## References

[1] Dutrillaux B. (1979). Chromosomal evolution in Primates: Tentative phylogeny from *Microcebus murinus* (Prosimian) to man. Qual. Life Res..

[2] Stanyon R., Rocchi M., Capozzi O., Roberto R., Misceo D., Ventura M., Cardone M.F., Bigoni F., Archidiacono N. (2008). Primate chromosome evolution: Ancestral karyotypes, marker order and neocentromeres. Chromosom. Res..

[3] Rocchi M., Archidiacono N., Schempp W., Capozzi O., Stanyon R. (2011). Centromere repositioning in mammals. Heredity.

[4] Dumas F., Mazzoleni S. (2017). Neotropical primate evolution and phylogenetic reconstruction using chromosomal data. Eur. Zool. J..

[5] Sineo L., Dumas F., Vitturi R., Picone B., Privitera O., Stanyon R. (2007). Williams-Beuren mapping in *Callithrix argentata*, *Callicebus cupreus* and *Alouatta caraya* indicates different patterns of chromosomal rearrangements in neotropical primates. J. Zool. Syst. Evol. Res..

[6] Picone B., Dumas F., Stanyon R., Lannino A., Bigoni F., Privitera O., Sineo L. (2008). Exploring evolution in Ceboidea (Platyrrhini, Primates) by Williams-Beuren Probe (HSA 7q11.23) chromosome mapping. Folia Primatol..

[7] Jauch A., Wienberg J., Stanyon R., Arnold N., Tofanelli S., Ishida T., Cremer T. (1992). Reconstruction of genomic rearrangements in great apes and gibbons by chromosome painting. Proc. Natl. Acad. Sci. USA.

[8] Dumas F., Stanyon R., Sineo L., Stone G., Bigoni F. (2007). Phylogenomics of species from four genera of New World monkeys by flow sorting and reciprocal chromosome painting. BMC Evol. Boil..

[9] Trifonov V., Stanyon R., Nesterenko A.I., Fu B., Perelman P.L., O’Brien P.C.M., Stone G., Rubtsova N.V., Houck M.L., Robinson T. (2008). Multidirectional cross-species painting illuminates the history of karyotypic evolution in Perissodactyla. Chromosom. Res..

[10] Dumas F., Sineo L. (2012). Chromosomal dynamics in platyrrhinae by mapping BACs probes. S. Biol. Res.

[11] Dumas F., Sineo L. (2014). The evolution of human synteny 4 by mapping sub-chromosomal specific probes in Primates. Caryologia.

[12] Dumas F., Sineo L. (2010). Chromosomal dynamics in Cercopithecini studied by Williams-Beuren probe mapping. Caryologia.

[13] Dumas F., Sineo L., Ishida T. (2015). Taxonomic identification of Aotus (*Platyrrhinae*) through cytogenetics|Identificazione tassonomica di Aotus (*Platyrrhinae*) mediante la citogenetica. J. Biol. Res..

[14] Dumas F., Cuttaia H., Sineo L. (2016). Chromosomal distribution of interstitial telomeric sequences in nine neotropical primates (*Platyrrhini*): Possible implications in evolution and phylogeny. J. Zool. Syst. Evol. Res..

[15] Mazzoleni S., Schillaci O., Sineo L., Dumas F. (2017). Distribution of interstitial telomeric sequences in primates and the pygmy tree shrew (*Scandentia*). Cytogenet. Genome Res..

[16] Mazzoleni S., Rovatsos M., Schillaci O., Dumas F. (2018). Evolutionary insight on localization of 18S, 28S rDNA genes on homologous chromosomes in Primates genomes. Comp. Cytogenet..

[17] Milioto V., Vlah S., Mazzoleni S., Rovatsos M., Dumas F. (2019). Chromosomal localization of 18S-28S rDNA and (TTAGGG)n sequences in two South African dormice of the genus *Graphiurus* (Rodentia: Gliridae). Cytogenet. Genome Res..

[18] Hruba M., Dvorak P., Weberova L., Subrt I. (2012). Independent coexistence of clones with 13q14 deletion at reciprocal translocation breakpoint and 13q14 interstitial deletion in chronic lymphocytic leukemia. Leuk. Lymphoma.

[19] Froenicke L. (2005). Origins of primate chromosomes—As delineated by Zoo-FISH and alignments of human and mouse draft genome sequences. Cytogenet. Genome Res..

[20] Murphy W.J., Larkin D.M., Der Wind A.E.-V., Bourque G., Tesler G., Auvil L., E Beever J., Chowdhary B.P., Galibert F., Gatzke L. (2005). Dynamics of mammalian chromosome evolution inferred from multispecies comparative Maps. Science.

[21] Ma J., Zhang L., Suh B.B., Raney B.J., Burhans R.C., Kent W.J., Blanchette M., Haussler D., Miller W. (2006). Reconstructing contiguous regions of an ancestral genome. Genome Res..

[22] Robinson T.J., Ruiz-herrera A., Froenicke L. (2006). Dissecting the mammalian genome—New insights into chromosomal evolution. Trends Genet..

[23] Kim J., Farré M., Auvil L., Capitanu B., Larkin D.M., Ma J., Lewin H.A. (2017). Reconstruction and evolutionary history of eutherian chromosomes. Proc. Natl. Acad. Sci. USA.

[24] Dunham A., Matthews L.H., Burton J., Ashurst J.L., Howe K.L., Ashcroft K.J., Beare D.M., Burford D.C., Hunt S.E., Griffiths-Jones S. (2004). The DNA sequence and analysis of human chromosome 13. Nature.

[25] Bailey J., Gu Z., Clark R.A., Reinert K., Samonte R.V., Schwartz S., Adams M.D., Myers E.W., Li P.W., Eichler E.E. (2002). Recent segmental duplications in the human genome. Science.

[26] Yunis J., Prakash O. (1982). The origin of man: A chromosomal pictorial legacy. Science.

[27] Cardone M.F., Alonso A., Pazienza M., Ventura M., Montemurro G., Carbone L., De Jong P.J., Stanyon R., D’Addabbo P., Archidiacono N. (2006). Independent centromere formation in a capricious, gene-free domain of chromosome 13q21 in Old World monkeys and pigs. Genome Boil..

[28] Alonso A., Mahmood R., Li S., Cheung F., Yoda K., Warburton P.E. (2003). Genomic microarray analysis reveals distinct locations for the CENP-A binding domains in three human chromosome 13q32 neocentromeres. Hum. Mol. Genet..

[29] Scardino R., Milioto V., Dumas F. (2018). Comparative Cytogenetics Allows the Reconstruction of Human Chromosome History: The Case of Human Chromosome 13. Cytogenetics-Past, Present and Further Perspectives.

[30] Small M.F., Stanyon R., Smith D.G., Sineo L. (1985). High-resolution chromosomes of rhesus macaques (*Macaca mulatta*). Am. J. Primatol..

[31] Murphy W.J., Eizirik E., Johnson W., Zhang Y.P., Ryder O.A., O’Brien S. (2001). Molecular phylogenetics and the origins of placental mammals. Nature.

[32] Maddison W.P., Maddison D.R.V. (2008). Mesquite: A modular system for evolutionary analysis. Biology.

[33] Koehler U., Bigoni F., Wienberg J., Stanyon R. (1995). Genomic reorganization in the concolor gibbon (*Hylobates concolor*) revealed by chromosome painting. Am. J. Phys. Anthropol..

[34] Hollatz M., Wienberg J., Müller S. (2003). Chromosomal phylogeny and evolution of gibbons (*Hylobatidae*). Qual. Life Res..

[35] Bigoni F., Houck M.L., Ryder O.A., Wienberg J., Stanyon R. (2004). Chromosome painting shows that *Pygathrix nemaeus* has the most basal karyotype among Asian colobinae. Int. J. Primatol..

[36] Bigoni F., Stanyon R., Wimmer R., Schempp W. (2003). Chromosome painting shows that the proboscis monkey (*Nasalis larvatus*) has a derived karyotype and is phylogenetically nested within Asian colobines. Am. J. Primatol..

[37] Nie W., Liu R., Chen Y., Wang J., Yang F. (1998). Mapping chromosomal homologies between humans and two langurs (*Semnopithecus francoisi* and *S. phayrei*) by chromosome painting. Chromosom. Res..

[38] Bigoni F., Koehler U., Stanyon R., Ishida T., Wienberg J. (1997). Fluorescence in situ hybridization establishes homology between human and silvered leaf monkey chromosomes, reveals reciprocal translocations between chromosomes homologous to human Y/5, 1/9, and 6/16, and delineates an X1X2Y1Y2/X1X1X2X2 sex-chromosome system. Am. J. Phys. Anthr..

[39] Bigoni F., Stanyon R., Koehler U., Morescalchi A.M., Wienberg J. (1997). Mapping homology between human and black and white colobine monkey chromosomes by fluorescent in situ hybridization. Am. J. Primatol..

[40] Stanyon R., Bruening R., Stone G., Shearin A., Bigoni F. (2004). Reciprocal painting between humans, De Brazza’s and patas monkeys reveals a major bifurcation in the *Cercopithecini phylogenetic* tree. Cytogenet. Genome Res..

[41] Finelli P., Stanyon R., Plesker R., Ferguson-Smith M., O’Brien P. (1999). Reciprocal chromosome painting shows that the great difference in diploid number between human and African green monkey is mostly due to non-Robertsonian fissions. Mamm. Genome.

[42] Moulin S., Gerbault-Seureau M., Dutrillaux B., Richard F.A. (2008). Phylogenomics of African guenons. Chromosom. Res..

[43] Wienberg J., Stanyon R., Jauch A., Cremer T. (1992). Homologies in human and *Macasa fuscata* chromosomes revealed by in situ suppression hybridization with human chromosome specific DNA libraries. Chromosoma.

[44] Neusser M., Stanyon R., Bigoni F., Wienberg J., Müller S. (2001). Molecular cytotaxonomy of New World monkeys (*Platyrrhini*)—Comparative analysis of five species by multi-color chromosome painting gives evidence for a classification of *Callimico goeldii* within the family of Callitrichidae. Cytogenet. Cell Genet..

[45] Gerbault-Serreau M., Bonnet-Garnier A., Richard F., Dutrillaux B. (2004). Chromosome painting comparison of *Leontopithecus chrysomelas* (Callitrichine, Platyrrhini) with man and its phylogenetic position. Chromosom. Res..

[46] Stanyon R., Bigoni F., Slaby T., Müller S., Stone G., Bonvicino C.R. (2004). Multi-directional chromosome painting maps homologies between species belonging to three genera of New World monkeys and humans. Chromosoma.

[47] Araújo N.P., Stanyon R., Pereira V.D.S., Svartman M. (2017). interspecific chromosome painting provides clues to the ancestral karyotype of the New World monkey genus aotus. J. Mamm. Evol..

[48] Ruiz-Herrera A., Garcia F., Aguilera M., Garcia M., Fontanals M.P. (2005). Comparative chromosome painting in Aotus reveals a highly derived evolution. Am. J. Primatol..

[49] Stanyon R., Garofalo F., Steinberg E.R., Capozzi O., Di Marco S., Nieves M., Archidiacono N., Mudry M. (2011). Chromosome painting in two genera of South American monkeys: Species identification, conservation, and management. Cytogenet. Genome Res..

[50] Stanyon R., Consigliere S., Müller S., Morescalchi A., Neusser M., Wienberg J. (2000). Fluorescence in situ hybridization (FISH) maps chromosomal homologies between the dusky titi and squirrel monkey. Am. J. Primatol..

[51] Garcia F., Nogues C., Ponsa M., Ruiz-Herrera A., Egozcue J., García M. (2000). Chromosomal homologies between humans and *Cebus apella* (Primates) revealed by ZOO-FISH. Mamm. Genome.

[52] Richard F., Lombard M., Dutrillaux B. (1996). ZOO-FISH Suggests a complete homology between human and *Capuchin Monkey* (*Platyrrhini*) euchromatin. Chromosome Res..

[53] Garcia F., Ruiz-Herrera A., Egozcue J., Ponsa M., Garcia M., García M. (2002). Chromosomal homologies between Cebusand Ateles (*Primates*) based on ZOO-FISH and G-banding comparisons. Am. J. Primatol..

[54] Stanyon R., Consigliere S., Bigoni F., Ferguson-Smith M., O’Brien P., Wienberg J. (2001). Reciprocal chromosome painting between a New World primate, the woolly monkey, and humans. Chromosom. Res..

[55] De Oliveira E.H.C., Neusser M., Pieczarka J.C., Nagamachi C.Y., Sbalqueiro I., Müller S. (2004). Phylogenetic inferences of Atelinae (*Platyrrhini*) based on multi-directional chromosome painting in *Brachyteles arachnoides*, *Ateles paniscus paniscus* and *Ateles b. marginatus*. Cytogenet. Genome Res..

[56] Morescalchi M.A., Schempp W., Consigliere S., Bigoni F., Wienberg J., Stanyon R. (1997). Mapping chromosomal homology between humans and the black-handed spider monkey by fluorescence in situ hybridization. Chromosom. Res..

[57] Consigliere S., Stanyon R., Koehler U., Arnold N., Wienberg J. (1999). In situ hybridization (FISH) maps chromosomal homologies between *Alouatta belzebul* (Platyrrhini, Cebidae) and other primates and reveals extensive interchromosomal rearrangements between howler monkey genomes. Am. J. Primatol..

[58] De Oliveira E.H.C., Neusser M., Figueiredo W.B., Nagamachi C.Y., Pieczarka J.C., Sbalqueiro I.J., Wienberg J., Müller S. (2002). The phylogeny of *howler monkeys* (Alouatta, Platyrrhini): Reconstruction by multicolor cross-species chromosome painting. Chromosom. Res..

[59] Finotelo L., Amaral P., Pieczarka J.C., De Oliveira E.H.C., Pissinati A., Neusser M., Müller S., Nagamachi C.Y. (2010). Chromosome phylogeny of the subfamily Pitheciinae (Platyrrhini, Primates) by classic cytogenetics and chromosome painting. BMC Evol. Boil..

[60] Barros R.M.S., Nagamachi C.Y., Pieczarka J.C., Rodrigues L.R.R., Neusser M., De Oliveira E.H.C., Wienberg J., Muniz J.A.P.C., Rissino J.D., Müller S. (2003). Chromosomal studies in *Callicebus donacophilus* pallescens, with classic and molecular cytogenetic approaches: Multicolour FISH using human and *Saguinus oedipus* painting probes. Chromosom. Res..

[61] Dumas F., Bigoni F., Stone G., Sineo L., Stanyon R. (2005). Mapping genomic rearrangements in titi monkeys by chromosome flow sorting and multidirectional in-situ hybridization. Chromosom. Res..

[62] Stanyon R., Bonvicino C.R., Svartman M. (2003). Chromosome painting in *Callicebus lugens*, the species with the lowest diploid number (*2n* = 16) known in primates. Chromosom. Res..

[63] Rodrigues L., Pieczarka J.C., Pissinati A., De Oliveira E.H.C., Rissino J.D.D., Nagamachi C.Y. (2011). Genomic mapping of human chromosome paints on the threatened masked Titi monkey (*Callicebus personatus*). Cytogenet. Genome Res..

[64] Araújo N., Santo A.A.D.E., Pereira V.D.S., Stanyon R., Svartman M. (2017). Chromosome painting in *Callicebus nigrifrons* provides insights into the genome evolution of Titi Monkeys and the ancestral callicebinae karyotype. Cytogenet. Genome Res..

[65] Cardone M.F., Ventura M., Tempesta S., Rocchi M., Archidiacono N. (2002). Analysis of chromosome conservation in *Lemur catta* studied by chromosome paints and BAC/PAC probes. Chromosom. Res..

[66] Warter S., Hauwy M., Dutrillaux B., Rumpler Y. (2004). Application of molecular cytogenetics for chromosomal evolution of the *Lemuriformes* (Prosimians). Cytogenet. Genome Res..

[67] Rumpler Y., Warter S., Hauwy M., Fausser J.-L., Roos C., Zinner D. (2008). Comparing chromosomal and mitochondrial phylogenies of sportive lemurs (Genus *Lepilemur*, Primates). Chromosom. Res..

[68] Stanyon R., Dumas F., Stone G., Bigoni F. (2006). Multidirectional chromosome painting reveals a remarkable syntenic homology between the greater galagos and the slow loris. Am. J. Primatol..

[69] Nie W., O’Brien P.C., Fu B., Wang J., Su W., Robinson T., Yang F., Ferguson-Smith M.A. (2006). Chromosome painting between human and lorisiform prosimians: Evidence for the HSA 7/16 synteny in the primate ancestral karyotype. Am. J. Phys. Anthr..

[70] Stanyon R., Koehler U., Consigliere S. (2002). Chromosome painting reveals that galagos have highly derived karyotypes. Am. J. Phys. Anthr..

[71] Nie W., Fu B., O’Brien P.C., Wang J., Su W., Tanomtong A., Volobouev V., Ferguson-Smith M., Yang F. (2008). Flying lemurs—The ’flying tree shrews’? Molecular cytogenetic evidence for a Scandentia-Dermoptera sister clade. BMC Boil..

[72] Muller S., Stanyon R., Ferguson-Smith M.A., Plesker R., Wienberg J., O’Brien P.C.M. (1999). Defining the ancestral karyotype of all primates by multidirectional chromosome painting between tree shrews, lemurs and humans. Chromosom. Res..

[73] Dumas F., Houck M., Bigoni F., Perelman P., Romanenko S., Stanyon R. (2012). Chromosome painting of the pygmy tree shrew shows that no derived cytogenetic traits link primates and scandentia. Cytogenet. Genome Res..

[74] Korstanje R., O’Brien P., Yang F., Rens W., Bosma A., Van Lith H., Van Zutphen L., Ferguson-Smith M. (1999). Complete homology maps of the rabbit (*Oryctolagus cuniculus*) and human by reciprocal chromosome painting. Cytogenet. Cell Genet..

[75] Graphodatsky A., Ferguson-Smith M., Stanyon R. (2012). A Short introduction to cytogenetic studies in mammals with reference to the present volume. Cytogenet. Genome Res..

[76] Graphodatsky A., Yang F., Dobigny G., Romanenko S.A., Biltueva L.S., Perelman P.L., Beklemisheva V.R., Alkalaeva E., Serdukova N.A., Ferguson-Smith M.A. (2008). Tracking genome organization in rodents by Zoo-FISH. Chromosom. Res..

[77] Stanyon R., Stone G., Garcia M., Froenicke L., García M. (2003). Reciprocal chromosome painting shows that squirrels, unlike murid rodents, have a highly conserved genome organization. Genomics.

[78] Li T., O’Brien P., Biltueva L., Fu B., Wang J., Nie W., Ferguson-Smith M., Graphodatsky A., Yang F. (2004). Evolution of genome organizations of *Squirrels* (Sciuridae) revealed by cross-species chromosome painting. Chromosom. Res..

[79] Yang F., Graphodatsky A., Li T., Fu B., Dobigny G., Wang J., Perelman P.L., Serdukova N.A., Su W., O’Brien P.C. (2006). Comparative genome maps of the pangolin, hedgehog, sloth, anteater and human revealed by cross-species chromosome painting: Further insight into the ancestral karyotype and genome evolution of eutherian mammals. Chromosom. Res..

[80] Nie W., Wang J., Su W., Wang Y., Yang F. (2009). Chromosomal rearrangements underlying karyotype differences between Chinese pangolin (*Manis pentadactyla*) and Malayan pangolin (*Manis javanica*) revealed by chromosome painting. Chromosom. Res..

[81] Breen M., Thomas R., Binns M.M., Carter N.P., Langford C.F. (1999). Reciprocal chromosome painting reveals detailed regions of conserved synteny between the karyotypes of the domestic dog (*Canis familiaris*) and human. Genomics.

[82] Yang F., O’Brien P., Milne B., Graphodatsky A., Solanky N., Trifonov V., Rens W., Sargan D.R., Ferguson-Smith M. (1999). A complete comparative chromosome map for the dog, red fox, and human and its integration with canine genetic maps. Genomics.

[83] Yang F., Graphodatsky A., O’Brien P.C.M., Colabella A., Solanky N., Squire M., Sargan D.R., Ferguson-Smith M.A. (2000). Reciprocal chromosome painting illuminates the history of genome evolution of the domestic cat, dog and human. Chromosom. Res..

[84] Cavagna P., Menotti A., Stanyon R. (2000). Genomic homology of the domestic ferret with cats and humans. Mamm. Genome.

[85] Perelman P.L., Graphodatsky A., Dragoo J.W., Serdyukova N.A., Stone G., Cavagna P., Menotti A., Nie W., O’Brien P.C.M., Wang J. (2008). Chromosome painting shows that skunks (Mephitidae, Carnivora) have highly rearranged karyotypes. Chromosom. Res..

[86] Yang F., Fu B., O’Brien P.C.M., Nie W., Ryder O.A., Ferguson-Smith M.A. (2004). Refined genome-wide comparative map of the domestic horse, donkey and human based on cross-species chromosome painting: Insight into the occasional fertility of mules. Chromosom. Res..

[87] Balmus G., Trifonov V., Biltueva L.S., O’Brien P.C., Alkalaeva E., Fu B., Skidmore J.A., Allen T., Graphodatsky A., Yang F. (2007). Cross-species chromosome painting among camel, cattle, pig and human: Further insights into the putative Cetartiodactyla ancestral karyotype. Chromosom. Res..

[88] Kulemzina A.I., Trifonov V., Perelman P.L., Rubtsova N.V., Volobuev V., Ferguson-Smith M.A., Stanyon R., Yang F., Graphodatsky A. (2009). Cross-species chromosome painting in Cetartiodactyla: Reconstructing the karyotype evolution in key phylogenetic lineages. Chromosom. Res..

[89] Volleth M., Heller K.-G., Pfeiffer R., Hameister H. (2002). A comparative ZOO-FISH analysis in bats elucidates the phylogenetic relationships between *Megachiroptera* and five microchiropteran families. Chromosom. Res..

[90] Mao X., Nie W., Wang J., Su W., Feng Q., Wang Y., Dobigny G., Yang F. (2008). Comparative cytogenetics of bats (Chiroptera): The prevalence of Robertsonian translocations limits the power of chromosomal characters in resolving interfamily phylogenetic relationships. Chromosom. Res..

[91] Mao X., Nie W., Wang J., Su W., Ao L., Feng Q., Wang Y., Volleth M., Yang F. (2007). Karyotype evolution in *Rhinolophus bats* (Rhinolophidae, Chiroptera) illuminated by cross-species chromosome painting and G-banding comparison. Chromosom. Res..

[92] Ye J., Biltueva L., Huang L., Nie W., Wang J., Jing M., Su W., Vorobieva N.V., Jiang X., Graphodatsky A. (2006). Cross-species chromosome painting unveils cytogenetic signatures for the Eulipotyphla and evidence for the polyphyly of Insectivora. Chromosom. Res..

[93] Volleth M., Müller S. (2006). Zoo-FISH in the European mole (*Talpa europaea*) detects all ancestral Boreo-Eutherian human homologous chromosome associations. Cytogenet. Genome Res..

[94] Yang F., Alkalaeva E.Z., Perelman P.L., Pardini A.T., Harrison W.R., O’Brien P.C.M. (2003). Reciprocal chromosome painting among human, aardvark, and elephant (*superorder Afrotheria*) reveals the likely eutherian ances-tral karyotype. PNAS.

[95] Kellogg M.E., Burkett S., Dennis T.R., Stone G., Gray B.A., McGuire P.M., Zori R., Stanyon R. (2007). Chromosome painting in the manatee supports Afrotheria and Paenungulata. BMC Evol. Boil..

[96] Ruiz-Herrera A., Robinson T. (2007). Chromosomal instability in Afrotheria: Fragile sites, evolutionary breakpoints and phylogenetic inference from genome sequence assemblies. BMC Evol. Boil..

[97] Robinson T., Fu B., Ferguson-Smith M.A., Yang F. (2004). Cross-species chromosome painting in the golden mole and elephant-shrew: Support for the mammalian clades Afrotheria and Afroinsectiphillia but not Afroinsectivora. R. Soc..

[98] Svartman M., Stone G., Page J.E., Stanyon R. (2004). A chromosome painting test of the basal eutherian karyotype. Chromosom. Res..

[99] Svartman M., Stone G., Stanyon R. (2006). The Ancestral eutherian karyotype is present in Xenarthra. PLoS Genet..

[100] Azevedo N.F., Svartman M., Manchester A., Moraes-Barros N., Stanyon R., Vianna-Morgante A.M. (2012). Chromosome painting in three-toed sloths: A cytogenetic signature and ancestral karyotype for Xenarthra. BMC Evol. Boil..

[101] Graphodatsky A., Perelman P.L., Sokolovskaya N.V., Beklemisheva V.R., Serdukova N.A., Dobigny G., O’Brien S., Ferguson-Smith M.A., Yang F. (2008). Phylogenomics of the dog and fox family (Canidae, Carnivora) revealed by chromosome painting. Chromosom. Res..

[102] Robinson T., Ruiz-Herrera A. (2008). Defining the ancestral eutherian karyotype: A cladistic interpretation of chromosome painting and genome sequence assembly data. Chromosom. Res..

[103] Byrne H., Rylands A.B., Carneiro J.C., Alfaro J.W., Bertuol F., da Silva M.N., Messias M., Groves C.P., Mittermeier R.A., Farias I. (2016). Phylogenetic relationship of the New World Titi monkeys (*Callicebus*): First appraisal of taxonomy based on molecular evidence. Front. Zool..

[104] Perelman P., Johnson W., Roos C., Seuanez H.N., Horvath J.E., Moreira M.A.M., Kessing B., Pontius J., Roelke M., Rumpler Y. (2011). A molecular phylogeny of living primates. PLoS Genet..

[105] Clemente I.C., Egozcue J., García M., García M. (1990). Evolution of the Simiiformes and the phylogeny of human chromosomes. Qual. Life Res..

