# Supplementary material for: Evolution of the Human Chromosome 13 Synteny: Evolutionary Rearrangements, Plasticity, Human Disease Genes and Cancer Breakpoints
Source: Genes (Basel). 2020 Apr 1;11(4):383. doi: 10.3390/genes11040383 (PMC7230465; doi:10.3390/genes11040383)
Supplement: Supplementary file 1 [file genes-11-00383-s001.zip › Supp Table 1 Map List OMIM.doc]

| **Gene Map List - on Chromosome 13** | | | |
| --- | --- | --- | --- |
| **Copyright (c) 1966-2018 Johns Hopkins University OMIM, data are provided for research purposes only.** | | | |
| **Cytogenetic location** | **Genomic coordinates (From NCBI/GRCh38)** | **Gene/Locus name** | **Phenotype** |
| 13pter-q12.13 |  | Corneal dystrophy, Fuchs endothelial, 2 (FECD2, FCD1) | Corneal dystrophy, Fuchs endothelial, 2 |
| 13q | 13:17700000-114364328 | Cholesterol level quantitative trait locus 1 (CLQTL1, CLF) | Cholesterol level QTL 1 |
| 13q12.11 | 13:18900000-22600000 | Attention deficit-hyperactivity disorder, susceptibility to, 6 (ADHD6) | Attention deficit-hyperactivity disorder, susceptibility to, 6 |
| 13q12-q14 | 13:18900000-54700000 | Dermatitis, atopic, 5 (ATOD5) | Dermatitis, atopic, susceptibility to, 5 |
| 13q12.11 | 13:18900000-22600000 | Fibrosis of extraocular muscles, congenital, 3C (CFEOM3C, FEOM4) | Fibrosis of extraocular muscles, congenital, 3C |
| 13q12 | 13:18900000-31600000 | Myocardial infarction, susceptibility to, 2 (MCI2) | Myocardial infarction, susceptibility to, 2 |
| 13q12.11 | 13:20138251-20161326 | Gap junction protein, alpha-3, 46kD (connexin 46)  (GJA3, CX46, CZP3, CAE3, CTRCT14) | Cataract 14, multiple types |
| 13q12.11 | 13:20187462-20192974 | Gap junction protein, beta-2, 26kD (connexin 26)  (GJA3, CX46, CZP3, CAE3, CTRCT14) | Deafness, autosoma recessive 1A  Deafness, autosoma dominante 3A  Vohwinkel syndrome  Bart-Pumphrey syndrome  Keratitis-ichthyosis-deafness syndrome  Hystrix-like icthyosis with deafness |
| 13q12.11 | 13:20221961-20232394 | Gap junction protein, beta-6 (connexin-30)  (GJB6, CX30, DFNA3B, DFNB1B, ECTD2, HED2) | Deafness, autosoma recessive 1B  Deafness, autosoma dominante 3B  Ectodermal dysplasia 2, Clouston type  Deafness, digenic GJB2/GJB6 |
| 13q12.11 | 13:21671075-21704500 | Fibroblast growth factor-9 (glia-activating factor)  (FGF9, SYNS3) | Multiple synostoses syndrome 3 |
| 13q12.12 | 13:22600000-24900000 | Myopia 20, autosoma dominante (MYP20) | Myopia 20, autosoma dominante |
| 13q12.12 | 13:23160307-23325164 | Sarcoglycan, gamma (35kD dystrophin-associated glycoprotein) (SGCG, LGMD2C, DMDA1, SCG3) | Muscular dystrophy, limb-girdle, type 2C |
| 13q12.12 | 13:23328822-23433727 | Sacsin (SACS, ARSACS) | Spastic ataxia, Charlevoix-Saguenay type |
| 13q12.12 | 13:23730188-23889447 | Mitochondrial intermediate peptidase  (MIPEP, COXPD31) | Combined oxidative phosphorylation deficiency 31 |
| 13q12.12-q12.13 | 13:24882273-24922888 | Centromeric protein J  (CENPJ, CPAP, MCPH6, SCKL4) | Microcephaly 6, primary, autosoma recessive  Seckel syndrome 4 |
| 13q12.13 | 13:25372010-26025850 | ATPase, class I, type 8A, member 2  (ATP8A2, ATPIB, CAMRQ4) | Cerebellar ataxia, mental retardation, and dysequilibrium syndrome 4 |
| 13q12.13 | 13:26132114-26223084 | RING finger protein-6 (RNF6) | Esophageal carcinoma, somatic |
| 13q12.2-q13 | 13:27200000-39500000 | Moebius syndrome (MBS, MBS1) | Moebius syndrome |
| 13q12.2 | 13:27251554-27256567 | Ribosomal protein L21 (RPL21, HYPT12) | Hypotrichosis 12 |
| 13q12.2 | 13:27620742-27667421 | Polymerase I, RNA, subunit D  (POLR1D, RPA16, RPAC2, TCS2) | Treacher Collins syndrome 2 |
| 13q12.2 | 13:27919981-27926313 | Pancreas/duodenum homeobox protein 1  (PDX1, IPF1, MODY4, PAGEN1) | Diabetes mellitus, type II, susceptibility to  Pancreatic agenesis 1  MODY, type IV |
| 13q12.2 | 13:28003273-28100591 | fms-related tyrosine kinase-3 (FLT3) | Leukemia, acute myeloid, somatic  Leukemia, acute myeloid, reduced survival in, somatic  Leukemia, acute myeloid, reduced survival in, somatic |
| 13q12.3 | 13:28659003-28678956 | Proteasome maturation protein (POMP, UMP1) | Keratosis linearis with ichthyosis congenita and sclerosing keratoderma |
| 13q12.3 | 13:30713477-30764427 | Arachidonate 5-lipoxygenase-activating protein  (ALOX5AP, FLAP) | Stroke, susceptibility to |
| 13q12.3 | 13:31199974-31332275 | Beta 3-glucosyltransferase (B3GLCT, B3GALTL, B3GTL) | Peters-plus syndrome |
| 13q13-q14.3 | 13:31600000-54700000 | Enuresis, nocturnal, 1 (ENUR1) | Enuresis, nocturnal, 1 |
| 13q13.1 | 13:31600000-33400000 | Hypophosphatemic rickets and hyperparathyroidism  (HPRHP) | Hypophosphatemic rickets and hyperparathyroidism |
| 13q13.1 | 13:32315479-32399671 | BRCA2 gene  (BRCA2, FANCD1, BROVCA2, GLM3, PNCA2) | Breast cancer, male, susceptibility to  Prostate cancer  Wilms tumor  Pancreatic cancer 2  Fanconi anemia, complementation group D1  Medulloblastoma  Glioblastoma 3  Breast-ovarian cancer, familial, 2 |
| 13q13.1 | 13:33016062-33066144 | Klotho (KL, KLOTHO) | Tumoral calcinosis, hyperphosphatemic  Coronary artery disease, susceptibility to |
| 13q13.3 | 13:34900000-39500000 | Inflammatory bowel disease 27 (IBD27) | Inflammatory bowel disease 27 |
| 13q13.3-q21 | 13:34900000-72800000 | Polydactyly, postaxial, type A5 (PAPA5) | Polydactyly, postaxial, type A5 |
| 13q13.3 | 13:36301637-36370179 | Spartin (SPG20) | Troyer syndrome |
| 13q13.3 | 13:36819201-36829602 | Regulatory factor X-associated protein  (RFXAP) | Bare lymphocyte syndrome, type II, complementation group D |
| 13q13.3 | 13:36844830-36920720 | Mothers against decapentaplegic, Drosophila, homolog of, 9 (MADH9, SMAD9, MADH6, PPH2) | Pulmonary hypertension, primary, 2 |
| 13q13.3 | 13:37000372-37009613 | Exosome component 8  (EXOSC8, OIP2, RRP43, PCH1C) | Pontocerebellar hypoplasia, type 1C |
| 13q13.3 | 13:38349770-38363006 | Ubiquitin-fold modifier 1 (UFM1, HLD14) | Leukodystrophy, hypomyelinating, 14 |
| 13q13.3 | 13:38687035-38887130 | FRAS1-related extracellular matrix protein 2  (FREM2, FRASRS2) | Fraser syndrome 2 |
| 13q14 | 13:39500000-54700000 | Body mass index quantitative trait locus 2 (BMIQ2) | Body mass index QTL2 |
| 13q14 | 13:39500000-54700000 | Bone mineral density quantitative trait locus 9 (BMND9) | Bone mineral density QTL 9 |
| 13q14 | 13:39500000-54700000 | Leukemia, chronic lymphocytic, susceptibility to, 2  (CLLS2, D13S25, DBM) | Leukemia, chronic lymphocytic, susceptibility to, 2 |
| 13q14 | 13:39500000-54700000 | Chromosome 13q14 deletion syndrome  (DEL13q14, C13DELq14) | Chromosome 13q14 deletion syndrome |
| 13q14 | 13:39500000-54700000 | Gastroesophageal reflux (GER) | Gastroesophageal reflux |
| 13q14.11 | 13:39500000-44600000 | Leprosy, susceptibility to, 6 (LPRS6) | Leprosy, susceptibility to, 6 |
| 13q14 | 13:39500000-54700000 | Rieger syndrome, type 2 (RIEG2, RGS2) | Rieger syndrome, type 2 |
| 13q14 | 13:39500000-54700000 | Spastic paraplegia 24, autosoma recessive (SPG24) | Spastic paraplegia 24, autosoma recessive |
| 13q14.11 | 13:39655626-39791664 | Component of oligomeric golgi complex 6  (COG6, COD2, KIAA1134, CDG2L, SHNS) | Shaheen syndrome  Congenital disorder of glycosylation, type IIl |
| 13q14.11 | 13:40555663-40666596 | Forkhead box O1A (forkhead in rhabdomyosarcoma)  (FOXO1A, FKHR) | Rhabdomyosarcoma, alveolar |
| 13q14.11 | 13:40789410-40812459 | Solute carrier family 25 (mitochondrial carrier), member 15 (ornithine transporter 1) (SLC25A15, ORNT1, HHH) | Hyperornithinemia-hyperammonemia-homocitrullinemia syndrome |
| 13q14.11 | 13:42562735-42608012 | Tumor necrosis factor ligand superfamily, member 11 (osteoprotegerin ligand)  (TNFSF11, OPGL, TRANCE, OPTB2) | Osteopetrosis, autosoma recessive 2 |
| 13q14.2-q14.1 | 13:46700000-46700000 | Autism, susceptibility to, 3 (AUTS3) | Autism susceptibility 3 |
| 13q14.2 | 13:46831541-46897075 | 5-hydroxytryptamine (serotonin) receptor-2° (HTR2A) | Schizophrenia, susceptibility to  Obsessive-compulsive disorder, susceptibility to  Major depressive disorder, response to citalopram therapy in  Anorexia nervosa, susceptibility to  Alcohol dependence, susceptibility to  Seasonal affective disorder, susceptibility to |
| 13q14.2 | 13:47942655-48001325 | Succinate-CoA ligase, ADP-forming, beta subunit (SUCLA2, MTDPS5) | Mitochondrial DNA depletion syndrome 5 (encephalomyopathic with or without methylmalonic aciduria) |
| 13q14.2 | 13:48037566-48052741 | Nucleoside diphosphate-linked moiety X motif 15 (NUDT15, MTH2, NUDT15D) | Thiopurines, poor metabolism of, 2 |
| 13q14.2 | 13:48233137-48262095 | Integral membrane protein 2B (BRI gene)  (ITM2B, BRI, ABRI, FBD, RDGCA) | Retinal dystrophy with inner retinal dysfunction and ganglion cell abnormalities  Dementia, familial Danish |
| 13q14.2 | 13:48303746-48481889 | Retinoblastoma-1 (RB1) | Small cell cancer of the lung, somatic  Retinoblastoma, trilateral  Retinoblastoma  Osteosarcoma, somatic  Bladder cancer, somatic |
| 13q14.2 | 13:48400896-48444703 | Lysophosphatidic acid receptor 6  (LPAR6, P2RY5, P2Y5, LAH3, ARWH1, HYPT8) | Woolly hair, autosoma recessive 1, with or without hypotrichosis  Hypotrichosis 8 |
| 13q14.2 | 13:49531943-49585586 | RCC1 domain- and BTB domain-containing protein 1  (RCBTB1, CLLD7, GLP, RDEOA) | Retinal dystrophy with or without extraocular anomalies |
| 13q14.3 | 13:50300000-54700000 | Stature quantitative trait locus 20 (STQTL20) | Stature QTL 20 |
| 13q14.3 | 13:50909677-50970461 | Ribonuclease H2, subunit B  (RNASEH2B, DLEU8, FLJ11712, AGS2) | Aicardi-Goutieres syndrome 2 |
| 13q14.3 | 13:51932668-52012129 | ATPase, Cu++ transporting, beta polypeptide  (ATP7B, WND) | Wilson disease |
| 13q14.3 | 13:52012386-52029647 | Alg11, S. cerevisiae, homolog of  (ALG11, KIAA1266, CDG1P) | Congenital disorder of glycosylation, type Ip |
| 13q21 | 13:54700000-72800000 | Ataxin 8 (ATXN8) | Spinocerebellar ataxia 8 |
| 13q21-q32 | 13:54700000-101100000 | Postaxial polydactyly, type A2 (PAPA2) | Postaxial polydactyly, type A2 |
| 13q21 | 13:54700000-72800000 | Specific language impairment QTL, 3 (SLI3) | Specific language impairment QTL, 3 |
| 13q21.2 | 13:59665582-60163984 | Diaphanous, Drosophila, homolog of, 3  (DIAPH3, DIA2, DRF3, AUNA1, NSDAN) | Auditory neuropathy, autosoma dominante, 1 |
| 13q21.33 | 13:70107212-70139752 | Ataxin 8 opposite strand (ATXN8OS, SCA8, KLHL1AS) | Spinocerebellar ataxia 8 |
| 13q21.3-q22.1 | 13:72782022-73016453 | Progesterone-induced blocking factor 1 (PIBF1, JBTS33) | Joubert syndrome 33 |
| 13q22 | 13:72800000-78500000 | Developmental dysplasia of the hip 1(DDH1) | Developmental dysplasia of the hip 1 |
| 13q22-q32 | 13:72,800,000-101,100,000 | Panic disorder syndrome 1 (PAND1) | Panic disorder syndrome 1 |
| 13q22.2 | 13:75,284,663-75,483,143 | TPC1 domain family, member 4 (TBC1D4, AS160, KIAA0603, NIDDM5) | Diabetes mellitus, noninsulin-dependent, 5 |
| 13q22.3 | 13:76,991,923-77,002,51 | CLN5 gene (CLN5) | Ceroid lipofuscinosis, neuronal, 5 |
| 13q22.3 | 13:77,895,480-77,975,722 | Endothelin receptor type B (EDNRB, HSCR2, ABCDS, WS4A) | ABCD syndrome,Waardenburg syndrome, type 4A, Hirschsprung disease, susceptibility to, 2 |
| 13q31-q33 | 13:78,500,000-109,600,000 | Nystagmus 4, congenital, autosoma dominante (NYS4) | Nystagmus 4, congenital, autosoma dominante |
| 13q31.1 | 13:80,335,975-80,341,114 | Sprouty, Drosophila, homolog of, 2 (SPRY2, IGAN3) | IgA nephropathy, susceptibility to, 3 |
| 13q31.1 | 13:83,877,204-83,882,392 | SLIT- and NTRK-like family, member 1  (SLITRK1, KIAA1910, TTM) | Trichotillomania,  Tourette syndrome  Deafness and myopia |
| 13q31.3-q32.1 | 13:89,400,000-97,500,000 | Mitral valve prolapse, myxomatous 3 (MMVP3) | Mitral valve prolapse, myxomatous 3 |
| 13q31.3 | 13:89,400,000-94,400,000 | Photoparoxysmal response 2 (PPR2) | Photoparoxysmal response 2 |
| 13q31.3 | 13:91,347,819-91,354,574 | Micro RNA 17 host gene  (MIR17HG, MIRH1, MIHG1, MIRHG1, C13orf25, FGLDS2) | Feingold syndrome 2 |
| 13q31.3-q32.1 | 13:93,226,807-94,408,019 | Glypican 6 (GPC6, OMIMD1) | Omodysplasia 1 |
| 13q32 | 13:94,400,000-101,100,000 | Keratoconus 7 (KTCN7) | Keratoconus 7 |
| 13q32 | 13:94,400,000-101,100,000 | Microcoria, congenital (chromosome 13q32 deletion syndrome) (MCOR, C13DELq32, DEL13q32) | Microcoria, congenital |
| 13q32 | 13:94,400,000-101,100,000 | Schizophrenia susceptibility locus, chromosome 13q-related (SCZD7) | Schizophrenia |
| 13q32 | 13:94,400,000-101,100,000 | Systemic lupus erythematosus, susceptibility to, 5 (SLEB5) | Systemic lupus erythematosus, susceptibility to, 5 |
| 13q32-q33 | 13:94,400,000-109,600,000 | Stature quantitative trait locus 4 (STQTL4) | Stature QTL 4 |
| 13q32.1 | 13:94,574,053-94,596,274 | TDP-glucose 4,6-dehydratase (TGDS, SDR2E1, CATMANS) | Catel-Manzke syndrome |
| 13q32.1 | 13:95,433,587-95,579,755 | Claudin 10 (CLDN10, OSPL, CPETRL3, HELIX) | HELIX syndrome |
| 13q32.1 | 13:95,677,138-95,794,988 | DnaJ, E. coli, homolog of, subfamily C, member 3 (protein kinase inhibitor p58) (DNAJC3, PRKRI, P58, ACPHD) | Ataxia, combined cerebellar and peripheral, with hearing loss and diabetes mellitus |
| 13q32.3 | 13:99,981,755-99,986,772 | ZIC family, member 2 (ZIC2, HPE5) | Holoprosencephaly 5 |
| 13q32.3 | 13:100,089,014-100,530,436 | Propionyl Coenzyme A carboxylase, alpha polypeptide  (PCCA) | Propionicacidemia |
| 13q32.3-q33.1 | 13:101,053,773-101,417,205 | Sodium leak channel, nonselective (NALCN, IHPRF1, CLIFAHDD) | Congenital contractures of the limbs and face, hypotonia, and developmental delay, Hypotonia, infantile, with psychomotor retardation and characteristic facies 1 |
| 13q33.1-q34 | 13:101,100,000-114,364,328 | Orofacial cleft 9 (OFC9) | Orofacial cleft 9 |
| 13q33.1 | 13:101,720,854-102,402,427 | Fibroblast growth factor-14 (FGF14, FHF4, SCA27) | Spinocerebellar ataxia 27 |
| 13q33.1 | 13:102,845,840-102,876,000 | Excision-repair, complementing defective, in Chinese hamster, number 5 (ERCC5, XPG, COFS3) | Cerebrooculofacioskeletal syndrome 3  Xeroderma pigmentosum, group G  Xeroderma pigmentosum, group G/Cockayne syndrome |
| 13q33.1 | 13:103,043,997-103,066,845 | Solute carrier family 10 (sodium/bile acid cotransporter family), member 2 (SLC10A2, NTCP2, PBAM) | Bile acid malabsorption, primary |
| 13q33.2 | 13:104,200,000-106,400,000 | Vesicoureteral reflux 5 (VUR5) | Vesicoureteral reflux 5 |
| 13q33.2 | 13:105,465,866-105,491,033 | D-amino acid oxidase activator (DAOA, G72) | Schizophrenia |
| 13q33.3 | 13:108,207,441-108,218,367 | Ligase IV, DNA, ATP-dependent, {Multiple myeloma, resistance to} (LIG4, LIG4S) | LIG4 syndrome |
| 13q34 | 13:109,600,000-114,364,328 | Deafness, autosoma dominante 33 (DFNA33) | Deafness, autosoma dominante 33 |
| 13q34 | 13:109,753,836-109,786,566 | Insulin receptor substrate 2 (IRS2) | Diabetes mellitus, noninsulin-dependent |
| 13q34 | 13:110,148,957-110,307,156 | Collagen IV, alpha-1 polypeptide (COL4A1, POREN1, HANAC, ICH, BSVD, RATOR) | Retinal arteries, tortuosity of  Angiopathy, hereditary, with nephropathy, aneurysms, and muscle cramps  Brain small vessel disease with or without ocular anomalies  Porencephaly1  Schizencephaly  Hemorrhage, intracerebral, susceptibility to |
| 13q34 | 13:110,307,266-110,513,208 | Collagen IV, alpha-2 polypeptide (COL4A2, POREN2, ICH) | Porencephaly 2  Hemorrhage, intracerebral, susceptibility to |
| 13q34 | 13:110,641,409-110,713,600 | Cysteinyl-tRNA synthetase 2 (CARS2, COXPD27) | Combined oxidative phosphorylation deficiency 27 |
| 13q34 | 13:110,712,622-110,721,073 | Inhibitor of growth 1 (ING1) | Squamous cell carcinoma, head and neck, somatic |
| 13q34 | 13:113,105,772-113,120,680 | Coagulation factor VII (F7) | Factor VII deficiency, Myocardial infarction, decreased susceptibility to |
| 13q34 | 13:113,122,798-113,149,528 | Coagulation factor X (F10) | Factor X deficiency |
| 13q34 | 13:113,155,863-113,172,385 | Protein Z (PROZ, PZ) | Protein Z deficiency |
| 13q34 | 13:113,645,770-113,735,66 | G protein-dependent receptor kinase 1 (rhodopsin kinase) (GRK1, RHOK, RK) | Oguchi disease-2 |
| 13q34 | 13:113,645,770-113,735,66 | Chromosome alignment-maintaining phosphoprotein 1  (CHAMP1, ZNF828, C13orf8, KIAA1802, MRD40) | Mental retardation, autosoma dominante 40 |
