# Supplementary material for: Evolution of the Human Chromosome 13 Synteny: Evolutionary Rearrangements, Plasticity, Human Disease Genes and Cancer Breakpoints
Source: Genes (Basel). 2020 Apr 1;11(4):383. doi: 10.3390/genes11040383 (PMC7230465; doi:10.3390/genes11040383)
Supplement: Supplementary file 1 [file genes-11-00383-s001.zip › Supp Table 2 Mittelman word.doc]

| **Distribution by band of the cancer breakpoints described in the Mitelman Database 2017** | | |
| --- | --- | --- |
| **Band** | **Entity** | **Morphology** |
| 13p13 | der(13)t(1;13)(q11;p13) | Malignant melanoma |
| 13p11 | der(13)t(1;13)(q12;p11) | Anaplastic large cell lymphoma, systemic type |
| 13p11 | der(13)t(2;13)(q11;p11) | Squamous cell carcinoma |
| 13p11 | der(13)t(6;13)(p11;p11) | Follicular lymphoma |
| 13p11 | dic(12;13)(p11;p11) | Acute lymphoblastic leukemia/lymphoblastic lymphoma, Acute myeloid leukemia, NOS |
| 13p11 | dic(13;22)(p11;p11) | Meningioma |
| 13p11 | t(1;13)(q12;p11) | Anaplastic large cell lymphoma, systemic type |
| 13p11 | t(13;15)(p11;q11) | Diffuse large B-cell lymphoma |
| 13p11 | t(2;13)(q11;p11) | Squamous cell carcinoma |
| 13p11 | t(6;13)(p11;p11) | Follicular lymphoma |
| 13q | del(13q) | Chronic myelomonocytic leukemia. Acute erythroleukemia (FAB type M6), Acute lymphoblastic leukemia/lymphoblastic lymphoma, Acute megakaryoblastic leukemia (FAB type M7), Acute monoblastic leukemia (FAB type M5), Acute monoblastic leukemia with differentiation (FAB type M5b), Acute monoblastic leukemia without differentiation (FAB type M5a), Acute myeloblastic leukemia with maturation (FAB type M2), Acute myeloblastic leukemia with minimal differentiation (FAB type M0), Acute myeloblastic leukemia without maturation (FAB type M1), Acute myeloid leukemia, NOS, Acute myelomonocytic leukemia (FAB type M4), Acute promyelocytic leukemia (FAB type M3), Adult T-cell lymphoma/leukemia (HTLV-1+), Bilineage or biphenotypic leukemia  Burkitt lymphoma/leukemia, Chronic lymphocytic leukemia, Chronic myeloid leukemia, aberrant translocation, Chronic myeloid leukemia, t(9;22), Chronic myeloproliferative disorder, NOS, Diffuse large B-cell lymphoma, Essential thrombocythemia Retinoblastoma, Follicular lymphoma, Idiopathic myelofibrosis, Mantle cell lymphoma.Mature B-cell neoplasm, NOS Monoclonal gammopathy of undetermined significance, Multiple myeloma, Mycosis fungoides/Sézary syndrome Myelodysplastic syndrome, NOS, Nodal marginal zone B-cell lymphoma, Nonneoplastic hematologic disorder/lesion,,Peripheral T-cell lymphoma, unspecified, Mesothelioma, Polycythemia vera, Refractory anemia, Refractory anemia with excess of blasts (FAB), Refractory anemia with ringed sideroblasts Refractory cytopenia with multilineage dysplasia, Atypical lipomatous tumor/atypical lipoma/well-differentiated liposarcoma, Lipoma, Spindle cell/pleomorphic lipoma |
| 13q11 | del(13)(q11) | Acute lymphoblastic leukemia/lymphoblastic lymphoma |
| 13q11 | del(13)(q11q14) | Acute erythroleukemia (FAB type M6), Acute lymphoblastic leukemia/lymphoblastic lymphoma. Acute myeloblastic leukemia without maturation (FAB type M1), Acute myeloid leukemia, NOS, Chronic lymphocytic leukemia |
| 13q11 | del(13)(q11q21) | Diffuse large B-cell lymphoma.Multiple myeloma |
| 12p11 | der(12)t(12;13)(p11;q11) | Acute erythroleukemia (FAB type M6). Acute lymphoblastic leukemia/lymphoblastic lymphoma |
| 13q11 | der(17)t(13;17)(q11;p11) | Chronic lymphocytic leukemia |
| 13q11 | der(3)t(3;13)(q29;q11) | Mantle cell lymphoma |
| 13q11 | t(12;13)(p11;q11) | Acute erythroleukemia (FAB type M6). Acute lymphoblastic leukemia/lymphoblastic lymphoma |
| 13q11 | t(13;17)(q11;p11) | Chronic lymphocytic leukemia |
| 13q11 | t(13;22)(q11;p11) | Adenocarcinoma |
| 13q11 | t(3;13)(q29;q11) | Mantle cell lymphoma |
| 13q11 | t(8;13)(p11;q11) | Chronic myeloproliferative disorder, NOS |
| 13q12 | del(13)(q12) | Acute lymphoblastic leukemia/lymphoblastic lymphoma. Bilineage or biphenotypic leukemia. Chronic lymphocytic leukemia Chronic myeloid leukemia, t(9;22) Lymphoplasmacytic lymphoma Multiple myeloma Lipoblastoma Spindle cell/pleomorphic lipoma. Waldenström macroglobulinemia |
| 13q12 | del(13)(q12q13) | Acute myeloblastic leukemia without maturation (FAB type M1) |
| 13q12 | del(13)(q12q14) | Acute erythroleukemia (FAB type M6). Acute lymphoblastic leukemia/lymphoblastic lymphoma. Acute megakaryoblastic leukemia (FAB type M7). Acute monoblastic leukemia (FAB type M5). Acute myeloblastic leukemia with maturation (FAB type M2). Acute myeloblastic leukemia with minimal differentiation (FAB type M0). Acute myeloblastic leukemia without maturation (FAB type M1), Acute myeloid leukemia, NOS, Acute myelomonocytic leukemia (FAB type M4), Acute promyelocytic leukemia (FAB type M3), Bilineage or biphenotypic leukemia Burkitt lymphoma/leukemia Chronic lymphocytic leukemia. Chronic myeloid leukemia, t(9;22), Diffuse large B-cell lymphoma Essential thrombocythemia Retinoblastoma Follicular lymphoma Idiopathic myelofibrosis Mantle cell lymphoma. Mature B-cell neoplasm, NOS Multiple myeloma Mycosis fungoides/Sézary syndrome Myelodysplastic syndrome, NOS, Nodal marginal zone B-cell lymphoma Nonneoplastic hematologic disorder/lesion Peripheral T-cell lymphoma, unspecified Mesothelioma, Polycythemia vera, Refractory anemia, Refractory anemia with excess of blasts (FAB), Refractory anemia with ringed sideroblasts |
|  |  |  |
| 13q12 | del(13)(q12q21) | Acute lymphoblastic leukemia/lymphoblastic lymphoma, Chronic lymphocytic leukemia Idiopathic myelofibrosis, Mature B-cell neoplasm, NOS Multiple myeloma, Refractory anemia with excess of blasts (FAB) |
| 13q12 | del(13)(q12q22) | Acute lymphoblastic leukemia/lymphoblastic lymphoma, Acute megakaryoblastic leukemia (FAB type M7), Acute monoblastic leukemia without differentiation (FAB type M5a) biphenotypic leukemia  Acute myeloid leukemia, NOS Chronic lymphocytic leukemia, Chronic myeloid leukemia, t(9;22), Chronic myelomonocytic leukemia, Chronic myeloproliferative disorder, NOS, Diffuse large B-cell lymphoma, Follicular lymphoma, Idiopathic myelofibrosis, Mature B-cell neoplasm, NOS, Multiple myeloma, Myelodysplastic syndrome, NOS, Polycythemia vera, Atypical lipomatous tumor/atypical lipoma/well-differentiated liposarcoma,.Lipoma |
| 13q12 | del(13)(q12q31) | Chronic lymphocytic leukemia |
| 13q12 | del(13)(q12q32) | Acute lymphoblastic leukemia/lymphoblastic lymphoma, Follicular lymphoma |
| 13q12 | der(13)t(7;13)(q11;q12) | Acute lymphoblastic leukemia/lymphoblastic lymphoma |
| 13q12 | der(17)t(13;17)(q12;p11) | Acute lymphoblastic leukemia/lymphoblastic lymphoma |
| 13q12 | der(9)t(9;13)(p13;q12) | Acute lymphoblastic leukemia/lymphoblastic lymphoma |
| 13q12 | t(1;13)(p12;q12) | Chronic lymphocytic leukemia |
| 13q12 | t(12;13)(p13;q12) | Acute lymphoblastic leukemia/lymphoblastic lymphoma |
| 13q12 | t(13;14)(q12;q32) | Acute lymphoblastic leukemia/lymphoblastic lymphoma |
| 13q12 | t(13;16)(q12;q22) | Wilms tumor |
| 13q12 | t(13;17)(q12;p11) | Acute lymphoblastic leukemia/lymphoblastic lymphoma |
| 13q12 | t(7;13)(q11;q12) | Acute lymphoblastic leukemia/lymphoblastic lymphoma |
| 13q12 | t(8;13)(p11;q12) | Bilineage or. Chronic myeloproliferative disorder, NOS |
| 13q12 | t(9;13)(p13;q12) | Acute lymphoblastic leukemia/lymphoblastic lymphoma |
| 13q13 | del(13)(q12q13) | Acute myeloblastic leukemia without maturation (FAB type M1) |
| 13q13 | del(13)(q13) | Acute lymphoblastic leukemia/lymphoblastic lymphoma, Acute myeloid leukemia, NOS Myelodysplastic syndrome, NOS, Plasma cell leukemia |
| 13q13 | del(13)(q13q14) | Acute myeloid leukemia, NOS, Chronic lymphocytic leukemia, Retinoblastoma, Follicular lymphoma, Multiple myeloma |
| 13q13 | del(13)(q13q21) | Acute lymphoblastic leukemia/lymphoblastic lymphoma, Acute myeloid leukemia, NOS Chronic lymphocytic leukemia, Follicular lymphoma, Idiopathic myelofibrosis, Mature B-cell neoplasm, NOS, Multiple myeloma, Refractory anemia with ringed sideroblasts, Refractory cytopenia with multilineage dysplasia |
| 13q13 | del(13)(q13q22) | Acute myeloblastic leukemia with maturation (FAB type M2), Acute myeloid leukemia, NOS Chronic lymphocytic leukemia, Diffuse large B-cell lymphoma, Retinoblastoma, Mature B-cell neoplasm, NOS, Multiple myeloma, Refractory anemia |
| 13q13 | del(13)(q13q31) | Acute lymphoblastic leukemia/lymphoblastic lymphoma, Acute megakaryoblastic leukemia (FAB type M7), Chronic lymphocytic leukemia, Diffuse large B-cell lymphoma,Follicular lymphoma, Idiopathic myelofibrosis, Multiple myeloma, Myelodysplastic syndrome, NOS Polycythemia vera, Refractory anemia with excess of blasts (FAB), Lipoma |
| 13q13 | del(13)(q13q32) | Acute lymphoblastic leukemia/lymphoblastic lymphoma, Acute megakaryoblastic leukemia (FAB type M7), Idiopathic myelofibrosis |
| 13q13 | der(1)t(1;13)(q43;q13) | Diffuse large B-cell lymphoma |
| 13q13 | t(1;13)(q43;q13) | Diffuse large B-cell lymphoma |
| 13q13 | t(9;13;22)(q34;q13;q11) | Chronic myeloid leukemia, aberrant translocation |
| 13q14 | del(13)(q11q14) | Acute erythroleukemia (FAB type M6), Acute lymphoblastic leukemia/lymphoblastic lymphom. Acute myeloblastic leukemia without maturation (FAB type M1), Acute myeloid leukemia, NOS, Chronic lymphocytic leukemia |
| 13q14 | del(13)(q12q14) | Acute erythroleukemia (FAB type M6), Acute lymphoblastic leukemia/lymphoblastic lymphoma, Acute megakaryoblastic leukemia (FAB type M7), Acute monoblastic leukemia (FAB type M5), Acute myeloblastic leukemia with maturation (FAB type M2), Acute myeloblastic leukemia with minimal differentiation (FAB type M0), Acute myeloblastic leukemia without maturation (FAB type M1), Acute myeloid leukemia, NOS, Acute myelomonocytic leukemia (FAB type M4), Acute promyelocytic leukemia (FAB type M3) Bilineage or biphenotypic leukemia Burkitt lymphoma/leukemia Chronic lymphocytic leukemia Chronic myeloid leukemia, t(9;22), Diffuse large B-cell lymphoma, Essential thrombocythemia, Retinoblastoma, Follicular lymphoma Idiopathic myelofibrosis, Mantle cell lymphoma Mature B-cell neoplasm, NOS, Multiple myeloma  Mycosis fungoides/Sézary syndrome, Myelodysplastic syndrome, NOS, Nodal marginal zone B-cell lymphoma, Nonneoplastic hematologic disorder/lesion Peripheral T-cell lymphoma, unspecified, Mesothelioma, Polycythemia vera, Refractory anemia, Refractory anemia with excess of blasts (FAB), Refractory anemia with ringed sideroblasts |
| 13q14 | del(13)(q13q14) | Chronic lymphocytic leukemia, Retinoblastoma, Follicular lymphoma, Multiple myeloma |
| 13q14 | del(13)(q14) | Acute lymphoblastic leukemia/lymphoblastic lymphoma Acute monoblastic leukemia without differentiation (FAB type M5a), Acute myeloid leukemia, NOS Adult T-cell lymphoma/leukemia (HTLV-1+), B-prolymphocytic leukemia Burkitt lymphoma/leukemia Chronic lymphocytic leukemia Chronic myeloid leukemia, aberrant translocation Retinoblastoma, Adenocarcinoma, Mature B-cell neoplasm, NOS, Multiple myeloma, Plasma cell leukemia, Osteosarcoma, NOS Alveolar rhabdomyosarcoma, Lipoma, Splenic marginal zone B-cell lymphoma |
| 13q14 | del(13)(q14q14) | Acute lymphoblastic leukemia/lymphoblastic lymphoma, Chronic lymphocytic leukemia Retinoblastoma, Monoclonal gammopathy of undetermined significance, Multiple myeloma Lipoma, Spindle cell/pleomorphic lipoma |
| 13q14 | del(13)(q14q21) | Acute lymphoblastic leukemia/lymphoblastic lymphoma, Chronic lymphocytic leukemia Retinoblastoma, Idiopathic myelofibrosis, Mantle cell lymphoma, Multiple myeloma |
| 13q14 | del(13)(q14q22) | Acute erythroleukemia (FAB type M6), Acute lymphoblastic leukemia/lymphoblastic lymphoma Acute myeloblastic leukemia with maturation (FAB type M2) Acute myeloid leukemia, NOS, Burkitt lymphoma/leukemia, Chronic lymphocytic leukemia, Chronic myeloid leukemia, t(9;22), Chronic myeloproliferative disorder, NOS, Diffuse large B-cell lymphoma Follicular lymphoma, Mantle cell lymphoma, Mature B-cell neoplasm, NOS, Multiple myeloma, Mycosis fungoides/Sézary syndrome, Lipoma, Spindle cell/pleomorphic lipoma |
| 13q14 | del(13)(q14q31) | Acute lymphoblastic leukemia/lymphoblastic lymphoma, Chronic lymphocytic leukemia Multiple myeloma |
| 13q14 | del(13)(q14q32) | Acute lymphoblastic leukemia/lymphoblastic lymphoma, Acute megakaryoblastic leukemia (FAB type M7), Acute myeloid leukemia, NOS, Bilineage or biphenotypic leukemia, Chronic lymphocytic leukemia, Multiple myeloma Polycythemia vera |
| 13q14 | del(13)(q14q33) | Acute lymphoblastic leukemia/lymphoblastic lymphoma, Retinoblastoma |
| 13q14 | del(13)(q14q34) | Acute lymphoblastic leukemia/lymphoblastic lymphoma, Acute myeloid leukemia, NOS Chronic myeloid leukemia, aberrant translocation, Multiple myeloma |
| 13q14 | der(13)t(1;13)(q21;q14) | Follicular lymphoma |
| 13q14 | der(13)t(5;13)(q11;q14) | Giant cell tumor of bone |
| 13q14 | der(1)t(1;13)(p36;q14) | Alveolar rhabdomyosarcoma |
| 13q14 | der(1)t(1;13)(q32;q14) | Diffuse large B-cell lymphoma |
| 13q14 | der(2)t(2;13)(q35;q14) | Alveolar rhabdomyosarcoma |
| 13q14 | der(2)t(2;13)(q36;q14) | Alveolar rhabdomyosarcoma |
| 13q14 | der(5)t(5;13)(p11;q14) | Osteosarcoma, NOS |
| 13q14 | der(8)t(8;13)(q24;q14) | Multiple myeloma |
| 13q14 | dup(13)(q21q14) | T-prolymphocytic leukemia |
| 13q14 | t(10;13)(q24;q14) | Chronic lymphocytic leukemia |
| 13q14 | t(10;13)(q26;q14) | Chronic lymphocytic leukemia |
| 13q14 | t(11;13)(q13;q14) | Follicular lymphoma |
| 13q14 | t(1;13)(p36;q14) | Follicular lymphoma, Refractory anemia with excess blasts-2, Alveolar rhabdomyosarcoma |
| 13q14 | t(1;13)(q21;q14) | Follicular lymphoma |
| 13q14 | t(1;13)(q23;q14) | Chronic lymphocytic leukemia |
| 13q14 | t(1;13)(q32;q14) | Diffuse large B-cell lymphoma |
| 13q14 | t(12;13)(p11;q14) | Acute lymphoblastic leukemia/lymphoblastic lymphoma, Chronic lymphocytic leukemia |
| 13q14 | t(12;13)(p13;q14) | Acute lymphoblastic leukemia/lymphoblastic lymphoma |
| 13q14 | t(12;13)(q15;q14) | Chronic lymphocytic leukemia |
| 13q14 | t(13;13)(q14;q22) | Chronic lymphocytic leukemia |
| 13q14 | t(13;14)(q14;q24) | Chronic lymphocytic leukemia |
| 13q14 | t(13;14)(q14;q32) | Chronic lymphocytic leukemia |
| 13q14 | t(13;15)(q14;q24) | Chronic lymphocytic leukemia |
| 13q14 | t(13;18)(q14;p11) | Chronic lymphocytic leukemia |
| 13q14 | t(13;18)(q14;q23) | Acute myeloid leukemia, NOS |
| 13q14 | t(13;19)(q14;p13) | Chronic lymphocytic leukemia |
| 13q14 | t(13;22)(q14;q13) | Chronic lymphocytic leukemia |
| 13q14 | t(2;13)(q35;q14) | Chronic lymphocytic leukemia, Alveolar rhabdomyosarcoma Rhabdomyosarcoma, NOS Alveolar rhabdomyosarcoma, Embryonal rhabdomyosarcoma |
| 13q14 | t(2;13)(q36;q14) | Alveolar rhabdomyosarcoma, Chronic lymphocytic leukemia, Alveolar rhabdomyosarcoma |
| 13q14 | t(3;13)(p26;q14) | Chronic lymphocytic leukemia |
| 13q14 | t(3;13)(q27;q14) | Burkitt lymphoma/leukemia |
| 13q14 | t(4;13)(q31;q14) | Chronic lymphocytic leukemia |
| 13q14 | t(5;13)(p11;q14) | Osteosarcoma, NOS, Giant cell tumor of bone |
| 13q14 | t(6;13)(p21;q14) | Chronic lymphocytic leukemia |
| 13q14 | t(6;13)(q15;q14) | Chronic lymphocytic leukemia |
| 13q14 | t(7;13)(p22;q14) | Chronic lymphocytic leukemia |
| 13q14 | t(8;13)(q12;q14) | Chronic lymphocytic leukemia |
| 13q14 | t(8;13)(q24;q14) | Chronic lymphocytic leukemia, Multiple myeloma |
| 13q14 | t(9;13;22)(q34;q14;q11) | Chronic myeloid leukemia, aberrant translocation |
| 13q14 | t(9;13)(q31;q14) | Chronic lymphocytic leukemia |
| 13q21 | del(13)(q11q21) | Diffuse large B-cell lymphoma, Multiple myeloma |
| 13q21 | del(13)(q12q21) | Acute lymphoblastic leukemia/lymphoblastic lymphoma, Chronic lymphocytic leukemia Idiopathic myelofibrosis, Mature B-cell neoplasm, NOS, Multiple myeloma, Refractory anemia with excess of blasts (FAB) |
| 13q21 | del(13)(q13q21) | Acute lymphoblastic leukemia/lymphoblastic lymphoma, Acute myeloid leukemia, NOS Chronic lymphocytic leukemia Follicular lymphoma Idiopathic myelofibrosis, Mature B-cell neoplasm, NOS, Multiple myeloma Refractory anemia with ringed sideroblasts Refractory cytopenia with multilineage dysplasia |
| 13q21 | del(13)(q14q21) | Acute lymphoblastic leukemia/lymphoblastic lymphoma, Chronic lymphocytic leukemia Retinoblastoma Idiopathic myelofibrosis, Mantle cell lymphoma, Multiple myeloma |
| 13q21 | del(13)(q21) | Acute lymphoblastic leukemia/lymphoblastic lymphoma, Acute myeloblastic leukemia without maturation (FAB type M1), Acute myeloid leukemia, NOS, Astrocytoma, grade III-IV/Glioblastoma Chronic lymphocytic leukemia Chronic myeloid leukemia, t(9;22) |
| 13q21 | del(13)(q21q22) | Acute monoblastic leukemia (FAB type M5) |
| 13q21 | del(13)(q21q31) | Acute monoblastic leukemia with differentiation (FAB type M5b), Diffuse large B-cell lymphoma, Multiple myeloma |
| 13q21 | dup(13)(q21q14) | T-prolymphocytic leukemia |
| 13q21 | dup(13)(q21q22) | Burkitt lymphoma/leukemia |
| 13q21 | dup(13)(q21q34) | Follicular lymphoma |
| 13q21 | t(9;13)(p21;q21) | Acute lymphoblastic leukemia/lymphoblastic lymphoma |
| 13q22 | del(13)(q12q22) | Acute lymphoblastic leukemia/lymphoblastic lymphoma, Acute megakaryoblastic leukemia (FAB type M7), Acute monoblastic leukemia without differentiation (FAB type M5a), Acute myeloid leukemia, NOS, Chronic lymphocytic leukemia, Chronic myeloid leukemia, t(9;22) Chronic myelomonocytic leukemia, Chronic myeloproliferative disorder, NOS, Diffuse large B-cell lymphoma, Follicular lymphoma, Idiopathic myelofibrosis, Mature B-cell neoplasm, NOS, Multiple myeloma, Myelodysplastic syndrome, NOS, Polycythemia vera, Atypical lipomatous tumor/atypical lipoma/well-differentiated liposarcoma, Lipoma |
| 13q22 | del(13)(q13q22) | Acute lymphoblastic leukemia/lymphoblastic lymphoma, Acute myeloblastic leukemia with maturation (FAB type M2), Acute myeloid leukemia, NOS, Chronic lymphocytic leukemia Diffuse large B-cell lymphoma Retinoblastoma, Mature B-cell neoplasm, NOS, Multiple myeloma Refractory anemia |
| 13q22 | del(13)(q14q22) | Acute erythroleukemia (FAB type M6), Acute lymphoblastic leukemia/lymphoblastic lymphoma, Acute myeloblastic leukemia with maturation (FAB type M2), Acute myeloid leukemia, NOS, Burkitt lymphoma/leukemia Chronic lymphocytic leukemia Chronic myeloid leukemia, t(9;22), Chronic myeloproliferative disorder, NOS, Diffuse large B-cell lymphoma Follicular lymphoma, Mantle cell lymphoma, Mature B-cell neoplasm, NOS, Multiple myeloma ,Mycosis fungoides/Sézary syndrome, Lipoma Spindle cell/pleomorphic, lipoma |
| 13q22 | del(13)(q21q22) | Acute monoblastic leukemia (FAB type M5) |
| 13q22 | del(13)(q22) | Acute lymphoblastic leukemia/lymphoblastic lymphoma, Adenocarcinoma, Burkitt lymphoma/leukemia, Chronic lymphocytic leukemia, Diffuse large B-cell lymphoma Squamous cell carcinoma, Mature B-cell neoplasm, NOS, Leiomyosarcoma, Lipoma, Adenocarcinoma |
| 13q22 | del(13)(q22q31) | Multiple myeloma |
| 13q22 | del(13)(q22q32) | Acute lymphoblastic leukemia/lymphoblastic lymphoma, Adult T-cell lymphoma/leukemia (HTLV-1+), Chronic lymphocytic leukemia, Multiple myeloma, Mesothelioma |
| 13q22 | der(10)t(10;13)(p15;q22) | Acute lymphoblastic leukemia/lymphoblastic lymphoma |
| 13q22 | dup(13)(q21q22) | Burkitt lymphoma/leukemia |
| 13q22 | t(10;13)(p15;q22) | Acute lymphoblastic leukemia/lymphoblastic lymphoma |
| 13q22 | t(13;13)(q14;q22) | Chronic lymphocytic leukemia |
| 13q31 | del(13)(q12q31) | Chronic lymphocytic leukemia |
| 13q31 | del(13)(q13q31) | Acute lymphoblastic leukemia/lymphoblastic lymphoma, Acute megakaryoblastic leukemia (FAB type M7), Chronic lymphocytic leukemia, Diffuse large B-cell lymphoma, Follicular lymphoma Idiopathic myelofibrosis, Multiple myeloma, Myelodysplastic syndrome, NOS Polycythemia vera, Refractory anemia with excess of blasts (FAB Lipoma) |
| 13q31 | del(13)(q14q31) | Acute lymphoblastic leukemia/lymphoblastic lymphoma, Chronic lymphocytic leukemia Multiple myeloma |
| 13q31 | del(13)(q21q31) | Acute monoblastic leukemia with differentiation (FAB type M5b), Diffuse large B-cell lymphoma, Multiple myeloma |
| 13q31 | del(13)(q22q31) | Multiple myeloma |
| 13q31 | del(13)(q31) | Acute myeloblastic leukemia with maturation (FAB type M2), Follicular lymphoma |
| 13q31 | del(13)(q31q33) | Diffuse large B-cell lymphoma |
| 13q31 | dup(13)(q31q33) | Burkitt lymphoma/leukemia |
| 13q32 | del(13)(q12q32) | Acute lymphoblastic leukemia/lymphoblastic lymphoma, Follicular lymphoma |
| 13q32 | del(13)(q13q32) | Acute megakaryoblastic leukemia (FAB type M7), Idiopathic myelofibrosis |
| 13q32 | del(13)(q14q32) | Acute lymphoblastic leukemia/lymphoblastic lymphoma, Acute megakaryoblastic leukemia (FAB type M7), Acute myeloid leukemia, NOS, Bilineage or biphenotypic leukemia Chronic lymphocytic leukemia Multiple myeloma, Polycythemia vera |
| 13q32 | del(13)(q22q32) | Acute lymphoblastic leukemia/lymphoblastic lymphoma, Adult T-cell lymphoma/leukemia (HTLV-1+), Chronic lymphocytic leukemia, Multiple myeloma, Mesothelioma |
| 13q32 | del(13)(q32) | Adult T-cell lymphoma/leukemia (HTLV-1+), Chronic lymphocytic leukemia Follicular lymphoma, Adenocarcinoma |
| 13q32 | der(13)t(1;13)(q21;q32) | Follicular lymphoma |
| 13q32 | t(1;13)(q21;q32) | Follicular lymphoma |
| 13q32 | t(X;13)(q13;q32) | Chronic myeloid leukemia, t(9;22) |
| 13q33 | del(13)(q14q33) | Acute lymphoblastic leukemia/lymphoblastic lymphoma, Retinoblastoma |
| 13q33 | del(13)(q31q33) | Diffuse large B-cell lymphoma |
| 13q33 | dup(13)(q31q33) | Burkitt lymphoma/leukemia |
| 13q34 | del(13)(q14q34) | Acute lymphoblastic leukemia/lymphoblastic lymphoma, Acute myeloid leukemia, NOS Chronic myeloid leukemia, aberrant translocation, Multiple myeloma |
| 13q34 | der(13)t(1;13)(q12;q34) | Multiple myeloma |
| 13q34 | der(13)t(1;13)(q21;q34) | Burkitt lymphoma/leukemia, Follicular lymphoma |
| 13q34 | der(13)t(1;13)(q23;q34) | Burkitt lymphoma/leukemia |
| 13q34 | der(13)t(7;13)(q21;q34) | Burkitt lymphoma/leukemia |
| 13q34 | dup(13)(q21q34) | Follicular lymphoma |
| 13q34 | t(10;13)(q24;q34) | Follicular lymphoma |
| 13q34 | t(1;13)(q12;q34) | Multiple myeloma |
| 13q34 | t(1;13)(q21;q34) | Burkitt lymphoma/leukemia, Follicular lymphoma |
| 13q34 | t(1;13)(q23;q34) | Burkitt lymphoma/leukemia |
| 13q34 | t(7;13)(q21;q34) | Burkitt lymphoma/leukemia |
